# NKX2-5 congenital heart disease mutations show diverse loss and gain of epigenomic, biochemical and chromatin search functions underpinning pathogenicity

**DOI:** 10.1101/2025.06.20.659510

**Published:** 2025-06-20

**Authors:** Alexander O. Ward, Nicole Schonrock, Alex J. McCann, Sabrina K. Phanor, Kian Hong Kock, Jesse V. Kurland, Fujian Wu, Nicholas J. Murray, James Walshe, Dimuthu Alankarage, Sally L Dunwoodie, Frederic A. Meunier, Mathias Francois, Martha L. Bulyk, Mirana Ramialison, Richard P. Harvey

**Affiliations:** 1 Developmental and Stem Cell Biology Division, Victor Chang Cardiac Research Institute, Darlinghurst, Sydney, New South Wales, Australia; 2 School of Clinical Medicine, UNSW Sydney, Kensington, New South Wales, Australia; 3 Clem Jones Centre for Ageing Dementia Research, Queensland Brain Institute, The University of Queensland, St Lucia, Queensland, Australia; 4 Division of Genetics, Department of Medicine, Brigham and Women’s Hospital and Harvard Medical School, Boston, Massachusetts, USA; 5 Program in Biological and Biomedical Sciences, Harvard University, Cambridge, Massachusetts, USA; 6 Department of Molecular Biology, Max Planck Institute, Gottingen, Germany; 7 School of Biomedical Science, The University of Queensland, St. Lucia, Queensland, Australia; 8 Centenary Institute, University of Sydney and Royal Prince Alfred Hospital, Sydney, New South Wales, Australia; 9 Department of Pathology, Brigham and Women’s Hospital, and Harvard Medical School, Boston, Massachusetts, USA; 10 Australian Regenerative Medicine Institute, Monash University, Clayton, Victoria, Australia; 11 The Novo Nordisk Foundation Center for Stem Cell Medicine, reNEW Melbourne, Murdoch Children’s Research Institute, Parkville, VIC, Australia; Department of Paediatrics, Faculty of Medicine, Dentistry and Health Sciences, University of Melbourne, Parkville, VIC, Australia; 12 School of Biotechnology and Biomolecular Science, UNSW Sydney, Kensington, New South Wales, Australia

## Abstract

Congenital heart defects (CHD) occur in ~1% of live births, with both inherited and acquired mutations and environmental factors known to contribute to causation. However, network perturbations and epigenetic changes in CHD remain poorly characterised. We report an integrated functional-epigenomics approach to understanding CHD, focusing on the cardiac homeodomain (HD) family transcription factor NKX2-5, mutations in which cause diverse congenital heart structural and conduction defects. We selected twelve NKX2-5 CHD-associated variants affecting different residue classes - DNA base-contacting, backbone-contacting, helix-stabilizing residues of the homeodomain, and those affecting other conserved protein:protein interaction (PPI) domains. In HL-1 cardiomyocytes, we profiled DNA targets of NKX2-5 wild type (WT) and variant proteins genome-wide using DamID, their DNA binding affinity and specificity using comprehensive protein binding microarrays, and PPI with known NKX2-5 cofactors using yeast 2-hybrid assay. We also undertook deep profiling of chromatin search and binding dynamics using single molecule tracking. Variants showed highly diverse but also class-specific behaviours with a range of severities. All variants failed to bind many WT targets but retained binding to a subset of core cardiomyocyte-related WT NKX2-5 targets, as well as hundreds of unique “off-targets”, in part via a regulatory logic that included changes to DNA binding site specificity, homodimerization and lost or enhanced cofactor interactions. All variants tested showed altered chromatin search functions. Our data suggest that complex residue-by-residue scale epigenomic, biochemical and chromatin search perturbations, involving both loss- and gain-of-function, contribute to CHD phenotypes. These findings may inform precision molecular therapeutic approaches in patients with CHD.

## INTRODUCTION

Congenital heart disease (CHD) is the most common congenital abnormality in humans, occurring in ~0.5–1% of all live births and accounting for up to 10% of spontaneous terminations^1^. Clinical and genetic studies have suggested pleiotropic genetic modes for CHD pathogenicity, involving inherited and *de novo* damaging mutations, chromosomal abnormalities, copy number variations, modifying common variants and polygenic mutation burden, complicating CHD diagnosis and the criteria for clinical actionability^2–4^.

Causative variants for CHD have been identified in a diverse set of genes known to coordinate early cardiac development, including those for cardiac transcription factors (TFs) such as NKX2-5, TBX5, TBX20 and GATA4^5–9^, intercellular signalling intermediates such as NOTCH, NODAL, SMAD and RAS proteins, as well as cilial proteins^10–14^, epigenetic regulators^4,15–20^ and structural proteins^3^. Despite enormous advances in understanding the genetic basis of CHD, the precise mechanisms of causation, especially epigenetic mechanisms, remain poorly understood. TFs are of particular interest because they are the spatiotemporal and evolutionary drivers of gene regulatory networks (GRNs)^21^ and interact dynamically with enhancers through an embedded motif grammar hard-wired into the genome^22^. Enhancers integrate genome, cell state, positional memory and signalling information to guide development of diverse cell lineages and tissue morphologies, and regeneration^23–26^. TFs implement changes in chromatin structure and gene expression on second-by-second or minute-by-minute timescales through diverse epigenetic processes^24,27^, including engagement with the transcriptional apparatus^28^, interactions with RNA^29–31^, and facilitated chromatin search functions^32^.

Cardiac TFs interact directly with each other as well as with a host of other cofactors, and collectively their genes are recursively wired (many cross-regulatory interactions) into the network^21,33–37^. Mutations in cardiac developmental TFs lead to catastrophic effects on heart development. For example, homozygous *Nkx2-5* null embryos show lethal arrest of heart development at early heart looping stages due to profound failures of cardiac field, chamber, major vessel and conduction system differentiation and proliferation, and accompanying severe perturbations to GRNs^38–44^.

Heterozygous mutations within human *NKX2-5* are most commonly associated with atrial and ventricular septal defects and atrioventricular conduction block, and more rarely an assortment of other severe defects, including tetralogy of Fallot, hypoplastic left heart, atrioventricular septal defects and heterotaxy^6,45,46^. There is no obvious genotype-phenotype correlation for individual NKX2-5 variants^45^ similar to CHD-causing variants in other cardiac TFs such as *TBX5* and *GATA4*, which show overlapping phenotypic spectrums^47,48^. Lack of linear relationship between genotype and phenotype is a general feature of dominant mutant alleles^49^.

Most variants in cardiac TFs lie in conserved or known functional domains and often are predicted to confer loss-of-function (LOF) effects via haploinsufficiency, a form of genetic dominance^49^. However, the partial penetrance and expressivity of variants suggest complex influences on CHD outcomes, such as retained functionalities of variants and the impact of genetic background of individual carriers^15,16,50,51^. Furthermore, there may be increased stochasticity of network outputs when TFs act close to functional thresholds^49^, undermining network robustness and bistability^52^. In true LOF, variant proteins would be unstable or incapable of performing their normal function. Recent evidence, however, suggests pervasive gain-of-function (GOF) effects for dominant TF variants in normal and cancerous cells^33,37,53–60^, including changes in DNA binding specificity^61^. We have previously shown that a CHD-causing NKX2-5 variant and a synthetic variant completely lacking the homeodomain (HD) retain the ability to bind and regulate a subset of normal NKX2-5 WT targets, whilst also binding to many unique “off-targets” leading to their modified expression^33^. We hypothesised, therefore, that TF GOF can act hand-in-hand with LOF to disrupt and destabilise the cardiac GRNs and contribute to CHD^33^.

**Figure 1.**
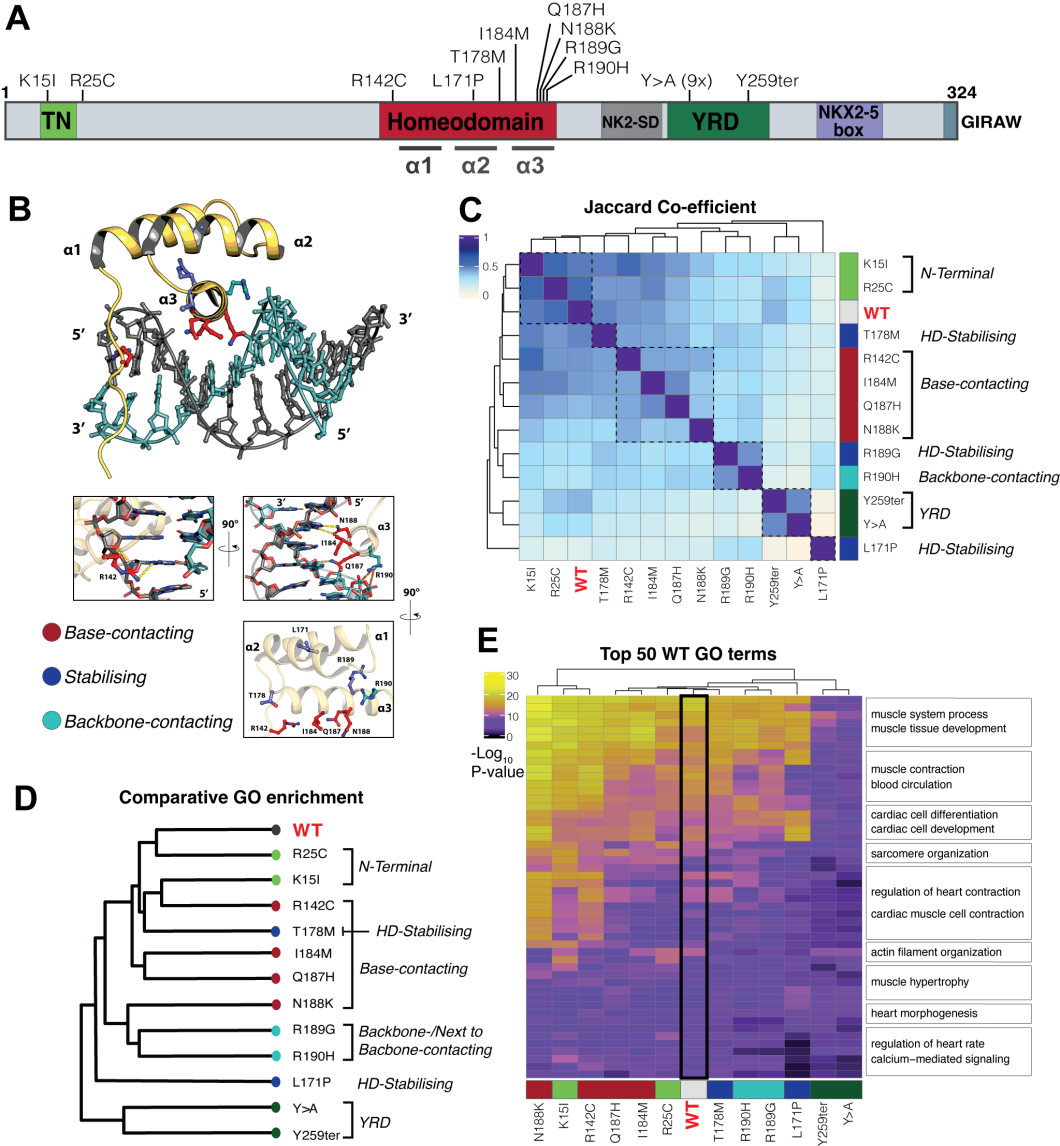
DamID reveals that Nkx2-5 allelic variants affect genomic targeting behaviours in a residue function specific manner (A) Schematic showing the amino acid positions of Nkx2-5 allelic variants. (B) Crystal structure of the Nkx2-5 WT Homeodomain (HD) with mutated residues highlighted by their functional class. (C) Degree of peak overlap of all WT and variant peaks measured using the Jaccard similarity co-efficient (between 0–1), with functional classes indicated and colour coded. (D) Unsupervised clustering of Nkx2-5 variants by the statistical similarity and log Odds Ratio scores of gene ontology (GO) terms, calculated using CompGO. (E) Significance values of the top 50 slimmed WT GO terms and corresponding significance for allelic variant, black indicates not significantly enriched.

We report here a broader analysis of NKX2-5 disease-causing variants identified through clinical genetics testing (Figure 1A). We selected 12 NKX2-5 variants affecting different functional classes of residue within the HD and other conserved PPI domains of NKX2-5, and determined DNA target genes, DNA binding specificity and affinity, and cofactor interactions. Because DNA binding domains encode chromatin search functions as well as DNA binding and PPIs^62^, for select variants we correlated findings with real-time measurements of chromatin search space behaviours using single molecule tracking (SMT). Whereas each variant showed unique properties, variant sub-classes targeted the genome in similar ways. All variants showed GOF effects, in some cases through a clear regulatory logic, including changes to DNA binding specificity, loss, retention or super-interaction with cofactors, and altered chromatin scanning functions. These unique genomic signatures reveal how different variant sub-classes perturb the cardiac GRN in different ways at single-residue scale, likely contributing to the clinical manifestation of CHD.

## MATERIALS AND METHODS

### Variant scoring with CADD, BayesDel and REVEL

Using the list of 143 pathogenic *NKX2-5* variants collated previously^45^, a VCF file was generated and uploaded to the Combined Annotation Dependent Depletion (CADD) online platform (version 1.6)^63^. The Raw CADD score was then extracted and plotted against the nucleotide variant position using custom R scripts. We also used the BayesDel and Rare Variant Ensemble Learner (REVEL) as implemented in Ensemble Variant Effect Predictor (VEP)^64,65^ to assess pathogenicity of NKX2-5 variants.

### Plasmids and Cloning

Sequences coding for cardiac TFs *Nkx2-5, Gata4, Tbx5, Tbx20* and *Hand1* were amplified from HL-1 cell cDNA, as described previously^33^. *Nkx2-5* and other cardiac TF cDNA were cloned into pCR8/GW/TOPO vectors with the pCR/GW/TOPO TA Cloning kit (Thermo Fisher, MA, USA). Mouse *Nkx2-5* variant sequences were generated using site-directed mutagenesis of *Nkx2-5* cDNA and Gateway-cloned into DamID lentiviral vectors pLgw-EcoDam-V5^66,67^, a gift from Bas van Steensel (Addgene plasmid #59210). Vectors used for lentivirus production were obtained from Prof Didier Trono: pMLDg/pRRE (Addgene #12251), pRSV-Rev (Addgene #12253), and pMD2.G (Addgene #12259)). For Yeast-2-Hybrid assays, *Nkx2-5* WT or variant cDNA was cloned from pCR8/GW/TOPO vectors into pGADT7 AD library vectors and *Nkx2-5* WT or other cardiac TF cDNA (listed above) were cloned into pGBKT7 DBD bait vectors (Clontech, Mountain View, USA). *Nkx2-5* WT or variant cDNA was Gateway-cloned into pDEST15-GST bacterial expression vectors, kindly gifted by Robert Shearer (Garvan Institute for Medical Research), for production of proteins used in protein binding microarray (PBM) experiments. Lastly, for single molecule tracking experiments, *Nkx2-5* WT or variant cDNA was cloned in frame with HaloTag (amplified from pENTR4-HaloTag (w876–1); Addgene plasmid #29644; gifted by Dr Eric Campeau), into the CMV-promoter driven vector, pCMX-GW1, kindly gifted by Dr Gavin Chapman (Victor Chang Cardiac Research Institute), using the NEBuilder HiFi DNA assembly kit (New England Biolabs (NEB), MA, USA).

### Cell Culture

The HL-1 cell-line was donated by Prof W C Claycomb (Department of Biochemistry and Molecular Biology, Louisiana State University, New Orleans, LA, USA)^68^. HL-1 cells were maintained in Claycomb Medium (Sigma-Aldrich, MO, USA; #51800C), supplemented with 0.1mmol/L norepinephrine, 100μg/mL Penicillin/Streptomycin, 2mmol/L L-Glutamine and 10% Foetal Bovine Serum. HEK293T, HeLa and Cos-7 cells were acquired from ATCC (Rockville, MD). These additional cell-lines were cultured in Dulbecco’s Modified Eagle Medium (DMEM) supplemented with 100μg/mL Penicillin/Streptomycin, 1% GlutaMAX (all Gibco) and 10% Foetal Bovine Serum (FBS) (Sigma Aldrich), at 37°C with 5% CO_2_, for growth and expansion. Culture conditions altered for experimental conditions will be detailed below.

### Liquid Yeast-2-Hybrid assay

Yeast-2-Hybrid assays were performed in liquid cultures as previously described^33^. 1 μg pGADT7-AD-Nkx2-5 (WT or variant), containing the Gal4 activation domain, and 1 μg pGBKT7-DBD-TF (NKX2-5, GATA4, TBX5, TBX20b or HAND1), containing the Gal4 DNA-binding domain, were co-transformed into chemically competent *S. Cerevisiae* (Clontech), using 240 μL PEG 3350 50%, 36 μL Li-Acetate 1.0 M, 25 μL Boiled SS-carrier DNA, 54 μL water. Double transformed cells were selected for growth on low stringency (-Leu/-Trp) selection plates at 30°C for 3 days, colonies were then picked and cultured in low-stringency liquid medium for a further 3 days at 30°C. Cultures were then selected for interactions in high stringency liquid medium (-Ade/-His/-Leu/Trp) at a 1:10 ratio of low stringency culture:high stringency medium and optical density (OD) of cultures was normalised. Fluoroscein Di-β-D-Galactopyranoside (FDG) was added to high-stringency medium to allow accurate fluorometric recordings of culture growth. Fluorescence was recorded at two time-points after after high stringency cultures (e.g. day 2 and day 3). Some interactions (NKX2-5 with HAND1 and GATA4) persisted beyond day 3 and were maintained until later time-points. Fluorescence recordings from time-point two (T_2_) were normalised to time-point one (T_1_).

### DNA-adenine methyltransferase identification (DamID)

DamID experiments were performed with modification of published protocols^67^ as described^33^. Briefly, confluent HL-1 cells were transduced with lentiviral vectors allowing undetectable expression of Dam-Nkx2-5 (WT or variant) fusion proteins driven by an uninduced *heat shock protein 68* promoter. After 40 hours, genomic DNA was extracted using a Gentra PureGene Cell kit (QIAGEN, Venlo, Netherlands), digested by *Dpn*I at 37°C for 6 hr, and amplified by ligation-mediated PCR. PCR products were further fragmented with DNase I at 24°C for 1 min, to lengths of between 200–2000 bps, and labelled and hybridised to Affymetrix mouse 1.0R promoter microarrays according to manufacturers’ instructions. Three independent DamID experiments were performed utilising biological triplicates.

### DamID bioinformatic analysis

#### Array processing, peak detection and gene assignment

Array processing, peak detection and gene assignment were performed as previously described^33^. Briefly, quality control was conducted using the ‘affy’ and ‘affyPLM’ R packages to assess probe-level distributions via RLE and NUSE plots, with outlier arrays excluded. Probe remapping to the mm9 genome (NCBI Build 37) was carried out using Starr, and array normalisation and peak calling were performed using CisGenome v2.0 with quantile normalisation and the TileMapv2 algorithm (minimum 8 probes, moving average ≥3.5). Peaks were assigned to genes using GREAT with “basal plus extension” settings (6.5 kb upstream, 2.5 kb downstream, max 100 kb), considering only non-coding regions covered by the array, with annotations retrieved from Ensembl API v66. Overlaps and distances to TSS were calculated using custom scripts and BEDTools.

#### Genomic footprinting

To evaluate similarity between transcription factor binding profiles across WT and variants, genomic footprinting was performed using the bedtools *bedr* R package (v1.0.7), which enabled efficient manipulation and comparison of genomic intervals in BED format. Peak files from each dataset were pre-processed to retain high-confidence regions within canonical chromosomes. Pairwise comparisons between all combinations of peak sets were conducted using the *jaccard* function in *bedr*, which computes the Jaccard index—defined as the size of the intersection divided by the size of the union of two sets of genomic intervals. This metric provides a scale-independent measure of similarity between binding profiles, capturing both shared and unique genomic occupancy. The resulting Jaccard indices were assembled into a symmetric similarity matrix representing all pairwise comparisons between samples or conditions. This matrix was visualised as a clustered heatmap using the *ComplexHeatmap* R package (v2.16.0)^69^. Hierarchical clustering with complete linkage and Euclidean distance was applied to group samples according to shared binding patterns. Annotations denoting NKX2-5 variant allele were included to aid interpretation, allowing for identification of conserved, divergent, or condition-specific regulatory footprints across the dataset.

#### GO analysis

Gene ontology (GO) enrichment analysis was performed using the *clusterProfiler* R package (v4.8.1) to identify biological processes significantly associated with genes linked to binding regions. Gene identifiers were first mapped to Entrez IDs using *org.Mm.eg.db*, and enrichment analysis was conducted using the *enrichGO* function with the *Biological Process* ontology. The entire mouse genome was used as the background to provide an unbiased enrichment context. To aid interpretability, GO term redundancy was reduced using the *simplify* function in *clusterProfiler*, which collapses semantically similar GO terms while retaining the most statistically significant representative. Enriched terms with adjusted p-values ≤ 0.05 were considered significant.

To support interpretation, visualisation of enriched GO terms was performed using built-in plotting functions including dot plots, bar plots, and enrichment maps. Where appropriate, gene concept networks were also generated to highlight shared gene-level contributions to multiple GO terms. For comparative enrichment analysis across multiple datasets or conditions, the *CompGO* R package^70^ was used. *CompGO* enables statistical comparison of GO term enrichment profiles by computing log odds ratios and associated confidence intervals between groups, facilitating direct assessment of whether specific biological processes are differentially enriched.

#### DNA motif discovery

Motif discovery and known motif enrichment analyses were performed using many of the same tools and procedures as previously described^33^. Briefly, *Trawler_standalone*^71^ was used for *de novo* motif discovery, with default parameters except where noted. Motif discovery was performed on repeat-masked sequences ±6.5 kb from the transcription start site of genes associated with binding peaks. To reduce redundancy among highly similar motifs, Trawler output was further processed using the *matrix clustering* module of *RSAT*^72^ which groups motifs into superfamilies based on semantic similarity scores, allowing for a more interpretable set of representative motif classes.

To complement the original motif discovery, additional *de novo* motif analysis was performed using the *HOMER* suite (v4.11)^73^. HOMER was run with default settings on peak-centred 200 bp sequences, and enrichment was calculated relative to a custom background set matched for GC content and length. *de novo* motifs identified by Trawler were further annotated using *TomTom* from the MEME Suite^74^, which performs motif-motif comparisons against curated databases (e.g., JASPAR) to identify putative transcription factor matches. Matches with q-values < 0.05 were considered significant.

Known motif enrichment analysis was carried out using *Clover* as previously described^33^, testing for overrepresentation of known transcription factor binding motifs in input peak sequences relative to a background composed of tiled regions from the Affymetrix Promoter 1.0R Array. Motifs with p-values < 0.05 were considered significantly enriched.

#### Network analysis

Protein–protein interaction networks were constructed using the *STRING* database (v12.0)^75^, which integrates evidence from curated databases, experimental data, gene co-expression, and text mining to infer both direct (physical) and indirect (functional) associations. Gene sets derived from peak-associated loci or enriched GO terms were submitted to the STRING web interface, restricted to *Mus musculus*, using a minimum interaction confidence score of 0.7 (high confidence). STRING analysis was used to identify significantly enriched interaction networks, with enrichment p-values calculated relative to a genome-wide background. Functional clustering within the interaction networks was performed using STRING’s built-in k-means clustering algorithm, revealing coherent modules based on shared network topology and biological function. Networks were exported from STRING and visualised using *Cytoscape* (v3.10.0)^76^, enabling interactive exploration and refinement of network topology. Functional clusters identified by STRING were preserved and annotated within Cytoscape to highlight distinct biological modules.

### Protein Binding Microarray (PBM) experiments

Glutathione S-transferase (GST)-Nkx2-5 (WT or variant) fusions were expressed from bacterial expression vectors, driven by a T7 promoter, using the PURExpress *In Vitro* Protein Synthesis kit (NEB), as per the manufacturer’s instructions. Molar concentrations of GST-Nkx2-5 fusion proteins were estimated using Western blotting and comparison against serial dilutions of recombinant GST (Sigma Aldrich, MO, USA). PBM experiments were performed as described^56^. Briefly, custom-designed, “all 10-mer” universal arrays in 8 × 60K, GSE format (Agilent Technologies; AMADID #030236) were double-stranded and used in PBM experiments with Alexa488-conjugated anti-GST antibody (Invitrogen A-11131), as described previously^77,78^. All proteins were assayed in at least duplicate at 200 nM final concentration in PBS-based binding and wash buffers. All three arrays analysed contained a Reference WT-NKX2-5 protein (WT1–3) to allow analysis against internal WT controls and cross-array comparisons. Specific analyses on the three arrays were as follows: v213: WT1, I184M, L171P, Q187H, R25C, R142C, T178M; v214: WT2, N188K, R189G, R190H, Y191C; v311, WT3, Y259ter. Arrays were scanned using a GenePix 4400A microarray scanner (Molecular Devices). At least 3 scans were taken for each slide at different photomultiplier tube (PMT) gain settings. Microarray data quantification, normalization, and motif derivation were performed essentially as described previously using the Universal PBM Analysis Suite and the Seed-and-Wobble motif-derivation algorithm^77^.

### Single Molecule Tracking (SMT) experiments

SMT experiments were carried out largely as described by McCann and colleagues^79^. Briefly, HeLa cells were seeded at a density of 1.5×10^5^ cells into 0.1% Gelatin-coated (Sigma Aldrich) glass-bottom 35mm FluoroDishes (World Precision Instruments, FL, USA) for 24 hours prior to transfection. Transient transfections were performed using XtremeGENE 9 transfection reagent (Roche, Switzerland), to introduce 1 μg plasmid DNA (pCMX-HaloTag-Nkx2-5 (WT or variant)), diluted in OptiMEM (Thermo Fisher) and complex was allowed to form, per manufacturer’s instructions. Transfections were incubated in FluoroBrite DMEM (referred to below as imaging medium) (Gibco), supplemented with 10% FBS and 1% GlutaMax for 24 hours at 37°C and under 5% CO_2_. Prior to imaging, cells were washed twice with Dulbecco’s PBS and labelled with 1nM JF549-HaloTag ligand (Promega, WI, USA) for 20 minutes in imaging medium at 37°C and under 5% CO_2_. The ligand was removed, cells were washed twice and fresh imaging medium was added. SMT images were acquired using an Elyra PALM/STORM (PS.1) super-resolution microscope with total internal reflection fluorescence (TIRF) architecture, using a 100x alpha Plan-Apochromat oil 1.46 NA objective and a BP 570–650/LP 750 filter cube (Zeiss, Germany). Samples were excited with an HR DPSS 531nm laser with a high-power TIRF filter and fluorescent signal was collected with the Andor iXon 897 EMCCD camera. Total power of the 531 nm laser line is 200 mW, at the low laser power used here, the power measured at the exit from the objective was 4.9 mW in epifluorescence mode, this value should be equivalent in oblique illumination mode. The oblique illumination angle used was 60.18°, equivalent to Highly Inclined and Laminated Optical sheet (HILO) illumination angle^80^. Imaging with low laser power and HILO illumination ensured that cells did not show any signs of phototoxicity or significant photobleaching during imaging. Two modes of SMT imaging were performed using this setup. Firstly, fast SMT at 50 Hz (20msec framerate) was performed to acquire 6000 frames. This enabled the tracking of single molecules with high temporal resolution (immobile and mobile populations). Secondly, slow SMT at 2 Hz (500msec framerate) was performed for 500 frames to remove background created by fast moving molecules and derive the bound lifetime of the immobile population. Final image resolution for both modes was 0.1μm × 0.1μm × 1μm. Image frame sizes were reduced to capture only a single nucleus, without changes to pixel size, preventing the need to post-crop images. Prior to capture, each nucleus was pre-bleached for 10–30 seconds, to reduce background fluorescence and the density of HaloTag-fusion molecules.

### SMT data analysis: fast SMT (20msec/50Hz)

#### SLIMfast tracking, SpotOn and diffusion coefficient calculation

Fast SMT image stacks were batch converted to tiff files using FIJI and analysed using similar pipelines to those published^32,81^ and MATLAB version 2015a. This pipeline uses the MATLAB script *SLIMfast.m*, which employs a two-step process to localise molecules, through a modified version of the multiple-target tracing (MTT) algorithm^82^. Similarly, to McCann *et al*.^79^, batch processing was performed with an error rate of 10^−6.5^, a detection box of 7 pixels, maximum number of iterations of 50, a termination tolerance of 10^−2^, a maximum position refinement of 1.5 pixels, an N.A. of 1.46, a PSF scaling factor of 1.35, and 20.2 counts per photon, an emission of 590 nm, a lag time of 20 ms and a pixel size 100 nm. Trajectory creation was performed using the maximum expected diffusion coefficient of 1.5 μm^2^/sec, determined from SpotOn analysis (above) and qualitative assessment of trajectories (i.e. presence of no/very few incorrectly localised molecules). Trajectories with less than 5 tracks were excluded and cells with significant divergence from normal distribution were removed as outliers using interquartile range thresholding. SLIMfast tracked data was then processed with custom MATLAB scripts to output log_10_ diffusion coefficient measurements and trajectory maps from ROIs of each cell (whole nucleus). Diffusion coefficient measurements for all cells were combined with another custom MATLAB script and plotted in GraphPad Prism.

#### TrackIt tracking and, kinetic modelling

Fast SMT image stacks were loaded directly into TrackIt v1.1^83^ using MATLAB version 2020b. TrackIt provides a fully-integrated interface for visualisation, processing and analysis of SMT^83^. Regions of interest (ROIs) were drawn around each nucleus to remove cytoplasmic and extracellular background signal. The nearest neighbour tracking algorithm was used with a threshold of 1.5, a tracking radius of 9 pixels, a minimum trajectory length of 5 tracks and a maximum of 2 gap frames between trajectories. Mean Squared Displacement (MSD) was fit with a power law function. 90% of tracked points and an offset of 0.5 were used for MSD fitting. Cells with significant divergence from normal distribution were removed as outliers using interquartile range thresholding. Fitted data was exported for principal component analysis (PCA) and dwell time survival function plotting, using custom R scripts. Raw tracked data for each cell was output for use in downstream analyses detailed below. Firstly, kinetic modelling was used to profile the diffusion coefficients of specific populations of molecules, and the fraction of total molecules in each population, using the SpotOn MATLAB package, developed by Hansen and colleagues^84^, run using MATLAB version 2020b. We used the 3-state/population model, which categorises molecules into: Bound, Slow-diffusing or Fast-diffusing states, allowing the capture of complex nuclear dynamics seen with many TFs. TrackIt tracked output files for individual cells were used in SpotOn, and a 3-state model was fit with default settings, except for the following changes: a framerate of 20ms, 6 timepoint delays considered, entire trajectories were used for fitting, 2 gap frames were allowed, 10 fitting iterations and the localisation error was fit from the data. In addition, the thresholds for min/max diffusion constants (in μm^2^/sec) were constricted as follows, to prevent incorrect fitting: Bound = 0.00001–0.1; Slow = 0.001–0.5; Fast = 0.2–5. Population data were then collated and plotted using custom R scripts.

### SMT data analysis: slow SMT (500msec/2Hz)

Slow SMT image stacks were batch converted to tiff files using FIJI and analysed using similar pipelines to those published^85^, run with MATLAB version 2015a. The pipeline uses the MATLAB script *SLIMfast.m*, which employs a two-step process to localise molecules, through a modified version of the multiple-target tracing (MTT) algorithm^82^. SLIMfast batch processing was performed with an error rate of 10^−6.5^, a detection box of 7 pixels, maximum number of iterations of 50, a termination tolerance of 10^−2^, a maximum position refinement of 1.5 pixels, an N.A. of 1.46, a PSF scaling factor of 1.35, and 20.2 counts per photon, an emission of 590 nm, a lag time of 500 ms and a pixel size 100 nm. Trajectory creation was performed using the maximum expected diffusion coefficient of 0.05 μm^2^/sec, determined from SpotOn analysis (above) and qualitative assessment of trajectories (i.e. presence of no/very few incorrectly localised molecules). Following this, a modified version of the *Calculate length_2fitting_v3.m* MATLAB script was used, taken from McCann and colleagues^79^, with script provided by Dr Zhe Liu (liuz11@janelia.hhmi.org). Briefly, the modified script batch calculates the lifetime of molecules for each cell and fits a one- and two-component exponential decay function, outputting decay curves. These decay curves provide the average dwell time for specific and non-specific fractions, and the ratio between both fractions. Cells with significant divergence from normal distribution following slow tracking analysis were removed as outliers using interquartile range thresholding. Dwell times for specific and non-specific fractions, and the ratio, were output directly and collated and plotted in GraphPad Prism.

## RESULTS

### Mutational landscape of NKX2-5

CHD variants are spread across the known conserved domains of NKX2-5 - trans-repressive *Tinman* (TN) domain, DNA-binding homeodomain (HD), NK2-Specific Domain (NK2SD), Tyrosine Rich Domain (YRD) and NKX2-5 Box^86,87^ (Figure 1A). To perform a detailed functional genomics analysis of NKX2-5 variant chromatin binding and search functions, we first selected natural variants previously associated with diverse CHD clinical phenotypes and assessed their predicted pathogenicity *in silico*. We scored 143 predicted pathogenic NKX2-5 variants identified in the literature and clinical databases^45^ using Combined Annotation Dependent Depletion (CADD)^88^ (Figure S1A). CADD uses an integrative approach based on over 60 complex genomic features, including sequence context, evolutionary constraint, and functional predictions. Variants within known conserved domains had higher pathogenicity scores compared to those falling between domains (inter-domain), whereas stop-gain NKX2-5 variants were predicted to be more deleterious than non-synonymous point mutations (Figure S1A). All HD variants were predicted to be highly pathogenic (far above the CADD likely pathogenic threshold of 2). However, both missense variants within specific HD helices (H1-H3) and those lying between helices (inter-HD) varied considerably with respect to clinical phenotype and CADD scores; thus, HD helical position likely carries distinct functional genomics outputs^61^. In a survey of human TF alleles, Barrera and colleagues^56^ showed that HD residues directly contacting DNA bases or backbone phosphates, or situated adjacent to these residues, were significantly enriched among Mendelian disease mutations. Therefore, for increased granularity on the functional genomic outputs of individual HD mutations, we classified HD variant residues according to their predicted HD functions as DNA-base-contacting (specific binding), DNA-backbone-contacting (non-specific binding), or HD-stabilising, rather than their overall helical position^56^ using the NKX2-5 HD crystal structure PDB 3RKQ as reference and visualised in cartoon form using PyMOL (Figure 1B)^89^.

A list of variants for in-depth functional profiling was chosen to include the following: 1) association with at least one or more CHD clinical phenotypes; 2) variants classified as pathogenic or likely pathogenic by the American College of Medical Genetics and Genomics (ACMG)^90^; 3) at least one HD variant affecting each significant sub-class of DNA-binding domain residue functionality (DNA-base-contacting, DNA-backbone-contacting, HD stabilising); 4) broad ranges of predicted pathogenicity scores using CADD; and 5) previously investigated *in vitro* (Figure 1A). Additionally included were: natural variant K15I located in the TN trans-repressor domain, chosen as a ‘mild’ variant^91^, and one previously characterised severe synthetic variant within the conserved YRD located downstream of the HD (Figure 1A), where all nine conserved tyrosine residues of the YRD were converted to alanine (Y>A)^86^, complementing the natural stop-gain variant in the YRD (Y259ter). A final list of twelve disease-causing variants was selected (Figure 1A) with clinical associations for the eleven natural variants shown in Figure S1B. Pathogenicity predictions for chosen missense variants using REVEL^65^ and BayesDel^64^ showed a similar spectrum of scores to CADD, largely concordant^61,92^ (Figure S1C). All variants localised to the nucleus in transfected Cos-7 cells, which lack endogenous NKX2-5 expression (Figure S2).

### Genome-wide target signature of NKX2-5 variants is linked to functional class of mutation

Profiling of genome-wide targets of NKX2-5 WT and variants was performed with DNA-adenine methyltransferase identification (DamID), a sensitive enzymatic method for detection of protein-DNA interactions (Figure S3A)^67,93–95^. DamID represents an alternative, but complementary technique to Chromatin Immunoprecipitation (ChIP)-based methods, avoiding problems due to poor quality ChIP antibodies and artefacts associated with chromatin crosslinking^96^. NKX2-5 WT or variant allele N-terminal fusions with bacterial DAM methylase were created and expressed from an uninduced *heat shock protein 68* (*hsp68*) promoter vector in the HL-1 cell line (derived from atrial cardiomyocytes), as reported previously^33,97,98^. Because DamID relies on enzymatic adenine methylation of accessible GATC targets by DAM, the low-level expression of DAM from the uninduced *hsp68* promoter has proven sufficient for DAM activity across the genome in the context of endogenous NKX2-5 WT protein, however, without disruption of GRNs due to over-expression (Figure S3A). As described in our previous work^33^, NKX2-5 WT and variant targets were detected by hybridizing GATC-me DNA fragments cleaved from the genome using the endonuclease *Dpn*1 to Affymetrix gene promoter microarrays covering ~10 kb upstream and downstream of the transcription start site (TSS), in triplicate. For NKX2-5 WT, we identified 1573 peaks with low false discovery rates (FDR) (<0.05 cutoff; Figure S3B–C). Approximately half of NKX2-5 variants led to a significant reduction in peak numbers, with the other half comparable to WT (Figure S3B). Despite this significant loss of targets, the distributions of WT and variant targets across genome features were broadly similar, suggesting no overt failure to correctly bind genomic targets among variants (Figure S3C–D). Target sites were mostly within 5 kb of the TSS (Figure S3D).

Following appropriate quality control^33^, we proceeded to compare the peak overlap of WT and variants using the Jaccard coefficient (JC) and saw concordance between variant classes and clear differences between classes (Figure 1C). The JC measures the similarity of two sets of peaks by calculating the ratio of the number of intersecting base-pairs between two peak sets to the total number of base-pairs within both peak sets, minus the intersection^99^. This statistical method showed that the peak footprints of variants within the same functional class overlapped extremely well with one another (higher JC) (Figure 1C). For example, the two NKX2-5 variants located at the N-terminus of the protein - K15I in the TN domain and R25C adjacent to the TN domain – classified by ACMG as Likely Pathogenic (LP) and a Variant of Uncertain Significance (VUS), respectively (light green; Figure 1A, C) - showed very similar peak footprints to NKX2-5 WT, and one another. In contrast, both natural (Y259ter) and synthetic (Y>A) variants in the YRD, a known protein-protein interaction (PPI) domain (dark green)^33^ showed highly similar peak footprints to one another, that were distinct from WT and all other variants (Figure 1C). These data reinforce the capacity of DamID to discern genome-wide regulatory signatures within and between NKX2-5 variant classes.

Of note, however, the genomic footprints of variants within the HD were much more nuanced, likely speaking to the underlying complexity of structure-function relationships between HD residues and the broad functionality of the HD overlapping as DNA-binding, PPI domain and chromatin search domain (Figure 1B)^32,33,37,56^. Comparison of target peak footprints for most DNA base-contacting residue variants showed that these clustered together, albeit showing a graded divergence from WT (Figure 1C, annotated in red). N188K was the most severe DNA base-contacting variant, in terms of regulatory divergences from WT, with R142C clustering closest to WT. Notably, the single DNA backbone-contacting variant chosen, R190H, was more severely affected than *all* DNA base-contacting variants. Furthermore, R190H clustered together with its adjacent HD-stabilising variant R189G, and these were highly distinct from WT and other HD stabilising variants. As such, R190H and R189G will henceforth be discussed together within the “backbone-contacting” class (Figure 1C), as they appear functionally similar across virtually all analyses. The two other variants in HD-stabilising residues, T178M and L171P, the latter a H2 helix-breaking variant^100^, showed highly divergent impacts, with T178M closer to WT and L171P the most distal to WT of all variants profiled. As noted, these variants likely reflect NKX2-5 HD structure and function in different ways.

Following assignment of unique genes to each DNA binding peak detected using GREAT^101^, WT and variant gene-sets were compared using the Spearman correlation coefficient with the resulting map showing highly similar groupings to that observed with JC analysis of peak overlaps, especially for the more severely affected variants (Supplementary Figure 4A). Hierarchical clustering and principal component analysis (PCA) of Gene Ontology (GO) terms associated with WT and variant gene-sets independently demonstrated the ability of DamID to discern target-site similarities and differences between variant subclasses (Figure 1D; Figure S4A–B). This granularity was further extended by visualisation of how cardiac muscle-related GO terms associated with NKX2-5 WT target genes compared to those of variant sub-classes (Figure 1E). Three variants, namely the N-terminal variant K15I (located in the TN repressor domain), and two base-contacting mutations, N188K and R142C, were associated with gain of cardiac muscle GO term significance, suggesting a broadening of cardiac-related targets compared to WT, noting that all three mutations had a similar number of target peaks to WT (Figure S3B). In contrast, R190H and R189G (backbone-contacting) variants, helix-breaking L171P variant and both YRD variants, were associated with a broad reduction of muscle GO term significance and a reduction in overall number of target peaks. This was true to a lesser degree for base-contacting variants I184M and Q187H. Thus, DamID analysis highlights different types of structure-function relationship within NKX2-5, evident at the level of both individual conserved domains and subdomains within the HD, some distinguished at single residue resolution, with implications for gene-regulatory network structure.

### NKX2-5 variants disrupt gene-regulatory logic in a functional domain-specific manner

**Figure 2.**
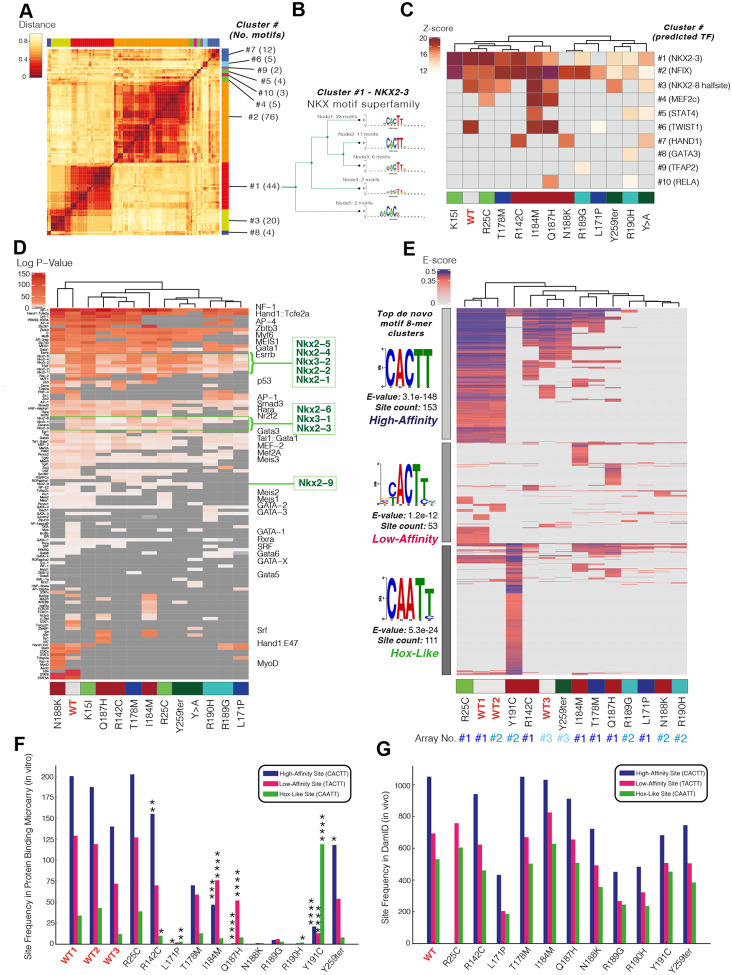
Disruption of DNA binding occurs in a residue function specific manner in Nkx2-5 variants (A) Distance matrix for all de novo discovered motifs identified using *Trawler* (values between 0–1) and motif clusters identified with RSAT matrix clustering. (B) Example of a reduced dendrogram for cluster #1, the NK2-type motif, highlighting the number of individual motifs discovered in each node (groups of similar motifs) and aggregate position weight matrix (PWM) for each node. (C) Significance (Z-score) of each de novo motif cluster identified with *Trawler*. (D) Significance (log p-value) of each known motif identified with *Clover*, with all motif names present on left and only cardiac-related motifs present on the right. (E) E-score distribution of all significantly bound 8-mers (above cut-off of 0.35) in 3 independent protein binding microarrays (PBM), with 3 major hierarchical clusters of motifs identified on the left (High-affinity, Low-affinity and Hox-like). (F) Frequency of three major detected 5-mer motifs (High-affinity, Low-affinity and Hox-like) in *in vitro* PBM 8-mers, across WT and all variants. (G) Frequency of three major detected 5-mer motifs (High-affinity, Low-affinity and Hox-like) in *in vivo* DamID peaks, across WT and all variants. Significance in (F and G) was calculated with a Two-sided Fisher’s exact test, * indicates p<0.05, ** indicates p<0.01, *** indicates p<0.001 and **** indicates p<0.0001 (not significant not indicated).

To analyse NKX2-5 gene regulatory control further, we profiled DamID peaks for transcription factor binding site (TFBS) motifs. Using Trawler^71,102^, a total of 175 unique *de novo* motifs were discovered across NKX2-5 WT and all variant peaks. Using RSAT to cluster these motifs semantically^72,102,103^ we identified 10 distinct sequence-related clusters (#1–10; Figure 2A). Tomtom^74^ was then employed to compare clusters of motifs against known motifs. In the aggregated DamID data, variations of the high affinity NKX2-5 binding motif (NKE) were prominent (cluster 1; Figure 2A–B). The Nuclear Factor 1 (NF-1) motif, which we have previously shown to be prevalent among NKX2-5 WT DamID target peaks^33^, was the dominant feature in cluster 2, more prevalently than the NKE itself (76 instances of aggregate NF-1 motif clusters versus 44 NKE clusters, respectively). Cluster 3 represented a NK2 HD half site (see below). Motifs of other TF family members occurred less often (2–12 instances across 8 separate *de novo* motif clusters). To visualise this graphically, we compared motif enrichment significance levels (Z-scores) in WT and variant peaks for all motif clusters (Figure 2B). For TN domain variant (K15I, R25C) peaks, there was highly significant over-representation of the high affinity NKE (dominant in cluster 1) and NF-1 motifs (NF1X cluster 2), in common with NKX2-5 WT, consistent with their similar DamID signatures. For HD mutations, the NKE enrichment score varied considerably, with R142C (base contacting) showing enrichment comparable to WT, whereas more severe mutations (e.g., base contacting N188K; HD stabilising L171P) showed no significant enrichment (Figure 2C), signifying highly divergent impacts of HD variant residues on NKE binding. The YRD variant also showed a low NKE enrichment score despite carrying an intact HD. Generally, there was partial concordance of enrichment significance for the NF-1 motif and NKX2-5 motifs. This suggests a close functional relationship between NKX2-5 and NF-1 factors on chromatin, comparable to that of NKX2-5 and other factors such as TBX5, GATA4, ISL1 and MEIS1^37,104,105^, and consistent with our previous finding that NKX2-5 and certain NF-1 isoforms are interacting cofactors^33^. Some variants showed strong Z-scores for NF-1 motifs and lower Z-scores for NKE-related motifs, e.g. N188K and R189G; for these, NKX2-5 may bind indirectly, potentially through interactions with NF-1 factors. The YRD variant showed a low Z-score for both NKE and NF-1 motifs, suggesting that the YRD is likely necessary for interaction of NKX2-5 with NF-1 proteins, as for ELK1/4 previously shown^33^. Cardiac TF family motifs MEF2 and HAND were also significantly enriched in certain variant target sets as a gain-of-function, e.g., the MEF2C motif showed highest Z-score for base contacting residue variants I184M and Q187H, which show similar target gene profiles (Figure 1C–D), noting that I184 and Q187 are both relatively weak DNA-base contacting residues^89^. Thus, NKX2-5 variant peaks show specific clustering based on their predicted TFBS motif composition.

As an alternative approach, we used Clover^106^ to directly assess known motif enrichment. As expected, we found strong enrichment of cardiac TF motifs (GATA, MEIS, HAND, MEF2, MYOD (bHLH), MYF6 (bHLH), and SRF) for NKX2-5 WT targets. Consistent with *de novo* motif Z-score allocations discussed above, we observed enrichment of NF-1 and NKX family motifs across variants to different degrees (NF-1 strongest for I184M and Q187H, as for *de novo* motif scoring) (2D). Other cardiac and muscle-related TF motifs were also notably enriched, albeit in mosaic patterns. Motifs associated with auxiliary co-factors (RARA, RXRA, AP-1, and p53) were enriched in WT and some variants.

### Analysis of NKX2-5 WT and variant motif binding in vitro and in vivo

We next sought to extend our understanding of NKX2-5 regulatory logic by investigating DNA-binding affinity and specificity for NKX2-5 WT and a selection of variants at single base pair resolution using *in vitro* protein binding microarrays (PBM) incubated with full-length *in vitro* translated NKX2-5 WT and variant glutathione S-transferase (GST) fusion proteins^77,107,108^. PBM allows in-depth and high-throughput analysis of the *in vitro* binding preference of proteins, including transcriptional and chromatin regulators, among all possible DNA sequences of lengths specified by the array design. The affinity of binding to DNA is reflected by enrichment (E)-scores, a rank-based metric that compares the distribution of k-mers across the full range of probe intensities, and therefore are robust to comparisons between PBM chips of different designs, scale and sensitivities^77,78^ (note that one of the three PBM arrays used, Array #3, comparing NKX2-5 WT with Y259ter, was less sensitive; see Figure 2E below). Initial analysis of position weight matrices (PWM) identified a palindromic motif composed of two NKX half sites (AAGT/ACTT) as the primary (most-significantly bound) 8-mer across three experiments, and the core NKE motif (CACTT) as the secondary 8-mer (Figure S5A–B); however, that the core NKE (secondary motif) was more significantly bound by WT NKX2-5 (i.e. higher average E-score^77^) than the half site palindrome (Figure S5C–D). All variants, except for R25C (located in the TN domain upstream of the HD), showed a reduction in binding to the NKE (Figure S5D). The pattern of binding of variants to the NKE and half site palindrome were similar, although Q187H (backbone-contacting) had higher affinity for the palindrome.

We next compared the most significantly bound 8-mer classes (E-score >0.35; false discovery rate <0.01^78^) between WT and variants (Figure 2E). Almost all variants, even those affecting domains outside of the HD, showed reduced binding affinity (fewer bound 8-mers, lower E-score), or altered binding specificity (deviation from the WT 8-mer binding patterns). Hierarchical clustering differentiated three major clusters of 8-mers present in our PBM data (grey bars in Figure 2E), which, when run through the MEME motif discovery tool, corresponded to the high-affinity NKE (classical NKX2-5 consensus binding sequence), low-affinity NKE (variation of the consensus sequence), and HOX-like homeodomain TFBS clusters. The observation that NKX2-5 binds to both the high-affinity consensus NKE site and HOX-like sites with lower affinity was shown previously by Electrophoretic Mobility Shift Assay (EMSA) and PBM^107,109^. Here, different variants displayed unique binding patterns with most, except R25C, showing a broad loss of binding across all 3 classes of 8-mers, but also some gain of binding to non-WT 8-mers. For the base-contacting Y191C variant, 8-mers were strikingly enriched in HOX-like sites (Figure 2E), consistent with our previous DamID findings^33^. This profound shift of motif preference is because Y191 is essential for the unique ability of helix 3 of the NKX2-5 HD to extend by 20 amino acids compared to other classes of HD proteins, allowing it to adopt an ordered state and high affinity binding to the NKE^110^. Base-contacting variants Q187H and I184M, and HD stabilising variant T178M, preferred binding to low-affinity NKE sites and/or unique sets of HOX-like sites. Proportional analysis of binding site distribution in PBM (high-affinity NKE; low-affinity NKE; HOX-like) (Figure 2F) validated the significance of Y191C binding to HOX-like sites and a shift in binding preference for I184M and Q187H from high- to low-affinity sites. Strikingly, R142C and Y259ter demonstrated the opposite trend, showing proportionally reduced binding to low-affinity NKE and HOX-like sites and a preference for higher-affinity NKE sites in comparison to WT.

We next assessed whether a comparable shift in binding site distribution was evident *in vivo*. All three classes of motif were found within NKX2-5 WT and variants target sites (Figure 2G). However, the proportionality changes found through PBM analysis *in vitro* were not seen in DamID peaks, suggesting that the observed *in vitro* shift is compensated for by the presence of endogenous WT NKX2-5 protein, transcriptional co-factors and/or supporting chromatin structure. Such compensation may contribute a degree of robustness to the GRN in the face of genetic perturbation.

### Influence of motif flanking 5’ and 3’ bases to DNA binding affinity in vitro and in vivo

We probed the impact of motif sequence on NKX2-5 regulatory logic by exploring the effects 5’ and 3’ bases flanking the NKE core site (CACTT). In principle, flanking based could impact DNA-binding specificity and affinity directly, or indirectly by altering chromatin search functions. In PBM experiments (Figure S5E), WT NKX2-5 showed a clear preference for a 5’-C and 3’-A flanking bases, whereas a 3’-T and several other flanking base conformations reduced binding affinity (Figure S5F–M). To investigate whether NKE flanking base preference was also observed *in vivo*, we analysed NKX2-5 WT DamID peaks for the occurrence of flanking sites (Figure S5N–P). GO term analysis of gene targets containing high- (**C**CACTT) and low-affinity flanking (CACTT**T**) motif sites in NKX2-5 WT DamID peaks revealed a significant functional segregation of these sites (Figure S5N–O). Genes close to high affinity sites were highly enriched in GO terms for cardiomyocyte developmental, structural and functional attributes (Figure S5N). Target genes close to low-affinity sites did not show a strong muscle signature and segregated with more peripheral dimensions of the cardiac GRN, such as metabolism and extracellular matrix, albeit non-significantly, suggesting that the high-low affinity site distribution form a part of the NKX2-5 core regulatory logic (Figure S5O). In addition, we found that most NKX2-5 variant peaks were more significantly enriched in low-affinity flanking sites than their high-affinity counterparts (Figure S5P), with exceptions being R25C, lying next to the N-terminal TN domain, and T178M, occurring in a HD stabilising residue, consistent with these variants having more WT-like target and GO term profiles (Figure 1C–E). These findings underscore that core flanking bases exert a significant influence on NKX2-5 WT binding affinity and TF regulatory logic, as has been found for other TFBS motifs^111–114^. They further show that the binding profile of most NKX2-5 variants, including those outside the HD, show a preference for lower affinity sites, likely influenced by changes in DNA binding preference *per se*, and in other specificity factors, such as co-factor interaction on DNA. Disturbances in the balanced regulation of core and peripheral elements of the cardiac GRN may be one mechanism underpinning the pathogenic effects of TF mutations.

### Variants within the YRD fail to bind cardiac regulatory genes due to loss of co-factor interactions

To further understand the structure-function consequences of NKX2-5 point mutations, we carried out a detailed differential peak analysis for all major variant classes (deep-dive data can be found in supplementary materials Figures S6–S12). We focused initially on the two variants within the YRD that showed highly similar features and severe network consequences, Y259ter is a stop-gain variant linked to multiple CHD phenotypes in humans, including atrial and ventricular septal defects, and double outlet right ventricle^45^. Y>A is a synthetic variant originally designed to probe the function of the YRD, in which all nine tyrosines of the YRD were substituted with alanines^86^. We first used CompGO, a tool for comparative GO term enrichment, combining the Jaccard coefficient and correlation analysis to statistically compare GO term enrichments between genes associated with WT and all YRD variant peaks^70^ (Figure S7A–B). Results indicated that genes associated with YRD mutations diverged from those of WT in terms of GO term enrichment, with Y259ter, which impacts only the last tyrosine among nine within the YRD, appearing less severe than Y>A.

**Figure 3.**
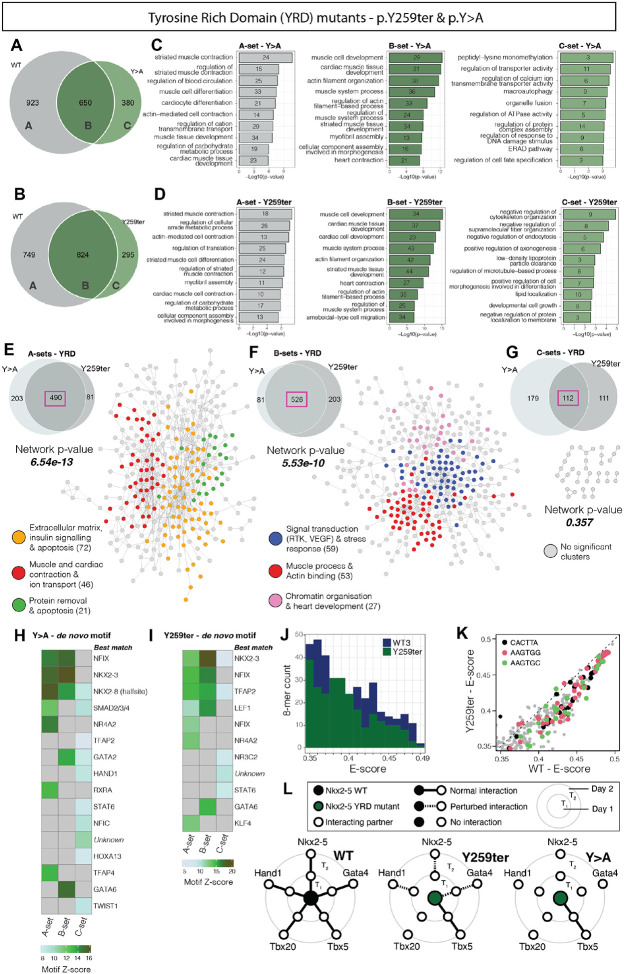
YRD variants result in the broad loss of specific cardiac regulatory programmes due to reduced homo- and hetero-dimerization (A) Overlap of WT and Y>A peaks. (B) Overlap of WT and Y259ter peaks. (C) Top 10 slimmed GO terms for targets of the A-, B- and C-sets from WT vs. Y>A (A). (D) Top 10 slimmed GO terms for targets of the A-, B- and C-sets from WT vs. Y259ter (B). (E) Overlap of genes lost (A-sets) by both YRD variants, from which a high-confidence STRING network was formed with 490 overlapping genes, showing highly significant network p-value, with cardiac network hubs highlighted in red. (F) Overlap of genes retained (B-sets) by both YRD variants, from which a high-confidence STRING network was formed with 526 overlapping genes, showing highly significant network p-value, with cardiac network hubs highlighted in red. (G) Overlap of genes gained (C-sets) by both YRD variants, from which a high-confidence STRING network was formed with 112 overlapping genes, showing no significant network enrichment or key hubs. (H) Significant Trawler de novo enriched motifs clustered to reduce redundancy for A-, B- and C-sets of Y>A variant. (I) Significant Trawler de novo enriched motifs clustered to reduce redundancy for A-, B- and C-sets of Y259ter variant. (J) Counts of significantly bound 8-mers (E > 0.35) from PBM, for WT (dark blue) and Y259ter (green). (K) Sequence specificity of significantly bound 8-mers (E > 0.35) from PBM, for WT against Y259ter, showing no change in specificity with Y259ter. (L) Interaction patterns of Nkx2-5 WT, Y259ter and Y>A variants with cardiac TFs Nkx2-5, Gata4, Tbx5, Tbx20 and Hand1, using a Yeast-two-hybrid (Y2H) protein-protein interaction assay. Y2H fluorescence intensities were normalised to Nkx2-5 WT at 2 different time-points (T_1_ and T_2_) and represented as normal, perturbed or absent interaction plots.

We next categorised WT and variant peaks for YRD variants into three subsets based on their overlap, as previously described^33^ (Figure 3A–B). A-sets were exclusively bound by WT, representing targets lost by variants; B-sets were bound by both WT and variants, representing targets retained by variants; and C-sets were uniquely bound by variants, which we previously termed “off-targets”. Comparing replicate experiments for NKX2-5 WT performed 2 years apart (n=3–4 per experiment), we have compellingly demonstrated that A sets (lost targets) and C sets (off-targets) are not due to experimental noise^34^. We performed GO term analysis on A-, B- and C-sets to gain deeper understanding of the functional implications of YRD mutations (Figure 3C–D). The A-sets (lost targets) showed over-representation of GO terms for biological processes pertaining to *striated muscle development* and *differentiation*, highlighting that YRD variants, even though retaining an intact DNA binding domain (HD), fail to bind elements of the core cardiac network (Figure 3C–D). Protein-protein interaction (PPI) network analysis at the gene level using STRING^115^ revealed that A-set genes of the two YRD variants displayed an extremely high degree of network significance/connectivity (Table S2). Indeed, comparison of both YRD variant A-set target genes showed a high degree of overlap (490 overlapping genes; 70% and 85% of total A-sets for Y>A and Y259ter, respectively) (Figure 3E). These overlapping lost (A-set) genes between Y>A and Y259ter had a highly significant STRING PPI networks enrichment p-value of 6.54e-13. Network analysis using Markov clustering^116^ revealed that cardiac-related genes, such as *Scn5a, Tbx5* and *Ryr2*, were at the core of a larger network (Figure 3E), again highlighting the systematic loss-of-function of cardiac network targets for YRD variants. Other key network clusters in YRD lost (A-set) targets included genes related to *extracellular matrix* and *insulin signalling* (*Irs1, Itga7, Timp3* and *Tgfb3*), as well as those related to *protein removal* and *apoptosis* (*Ubc, Ube1, Nfkb1* and *Bcl10*).

Within the substantial B-set genes for the two YRD variants (retained targets), we also observed significant enrichment of GO terms for biological processes related to *striated muscle development, differentiation* and *function*, evidently defining a distinct component of the muscle GRN to that detected in A-sets (Figure 3C–D and S6C–D). Interaction analysis of overlapping B-set genes between YRD variants showed a highly connected network (p-value of 5.53e-10) that indicated retention of a significant core of cardiac-related genes involved in adult muscle processes, such as actin binding (*Ttn, Myl1, Des* and *Actc1*), as well as embryonic gene signatures involved in heart development and chromatin organisation (*Tbx20, Smarca4* and *Ctcf*) (Figure 3F). The A- and B-set data show that YRD variants have reduced binding to a highly connected set of core cardiac network genes yet retain binding to other elements of the cardiac regulatory network.

There were also substantial numbers of C-set genes for both YRD variants (295/1119 targets for Y259ter; 380/1030 targets for Y>A) (Figure 3A–B). C-sets represent unique “off-targets” not bound by NKX2-5 WT. We, and others, have previously identified an underly logic for off-target selection by NKX2-5 and other cardiac TF variants, using DamID and ChIP, respectively^33,37,57^. However, despite high overlap of YRD variant C-set genes studied here, there was little or no network overlap at the GO term level and no cardiac-related terms were evident (Figure 3A–D and S6C–D). Furthermore, STRING network analysis revealed poor interconnectivity of target proteins in the C-sets of Y>A and Y259ter (Table S2), and in the overlap of C-sets (Figure 3G). Together, this suggests that, for YRD variants, the regulatory logic underpinning C-set off-target selection remains hidden (see below).

To probe regulatory logic further, *de novo* motif discovery was applied to YRD variant A-, B- and C-set target peaks (as applied above to an aggregate of WT and variant peaks; Figure 2A–B). For both YRD variants, A-set targets (lost targets) showed pronounced overrepresentation of NK2-class (NKE) and NF-1 binding sites, as well as other motifs including those for SMAD2/3/4, NR4A2, TFAP4, RXRA and KLF4 (Figure 3H–I). B-set targets also showed overrepresentation of NKE and NF-1 motifs, suggesting partial disruption of the capacity of variants to bind NKE and NF-1 motifs in core elements of the cardiac network. B-sets uniquely showed overrepresentation of GATA and LEF1 motifs compared to A-sets. C-set targets, which showed no obvious network interconnectivity or logic, nonetheless showed overrepresentation of motifs for TFAP2, STAT6, and HAND1, however, at lower Z-scores. This overall pattern was further corroborated using *de novo* motif analysis tool, Homer^73^, and known motif enrichment analysis tool, Clover^106^, which revealed significant differences between A- and B-sets, with fewer motifs in C-sets, confirming a clear regulatory logic for A- and B-sets but less so for C-sets (Figure S7E–H). In Clover analysis, some NKX and NF-1 motifs were retained in C-sets, but these are likely to be lower affinity. Overall, the Y259ter mutation showed little change in DNA-binding affinity or specificity compared to NKX2-5 WT, as shown from PBM data above for the high affinity NKE sites (Figure S5D) and as the distribution of all significant 8-mers as a function of E-scores (Figure 3J–K). These analyses powerfully highlight the importance for YRD variants of DNA-binding cofactor interactions in regulatory logic, as seen in the loss and retention of targets in DamID A- and B-sets, respectively, despite normal DNA-binding preference in PBM.

We have previously shown that the YRD has a key role in facilitating NKX2-5 homodimerization (previously thought to occur exclusively through the HD) and heterodimerization, demonstrated for TFs ELK1 and ELK4^33^. We therefore systematically profiled the homo- and heterodimerization capabilities of NKX2-5 WT and YRD variants using a liquid-based yeast 2-hybrid (Y2H) protein-protein interaction assay, exploring binding between NKX2-5 WT and cardiac cofactors GATA4, TBX5, TBX20 and HAND1, with data collected at two time points (day 1 (T_1_) and 2 (T_2_)) (Figure 3L and S6I). NKX2-5 WT demonstrated sustained or increasing interactions over time with itself and all tested cardiac TFs, known to interact directly with NKX2-5^33^. In contrast, both Y259ter and Y>A variants exhibited significantly disrupted or complete loss of interactions with NKX2-5 WT and cardiac co-factors, with the exception of TBX5, which is known to interact through the NKX2-5 HD^37^ (Figure 3L). These findings suggest that many of the core cardiac TFs interact with NKX2-5 through the YRD. Interestingly, the synthetic Y>A variant, in which all nine tyrosines of the YRD have been substituted for alanine^86^, showed more profound loss of interaction with NKX2-5 and cofactors compared to the natural Y259ter variant, which impacts only the last of the nine tyrosines. These results again reinforce that cofactor interactions via the YRD play a critical role in the selection of *in vivo* target-sites by NKX2-5 in the presence of an intact HD.

### Homeodomain DNA-backbone-contacting residue variants result in a broad loss of targets

**Figure 4.**
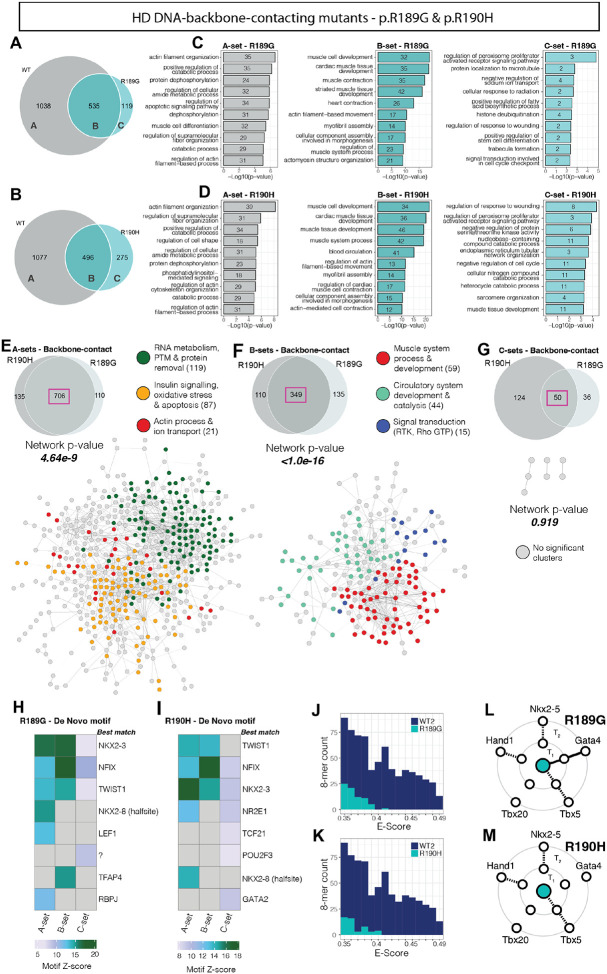
HD DNA-backbone-contacting variants broadly lose many target due to loss of DNA-binding and homo- and hetero-dimerization (A) Overlap of WT and R189G peaks. (B) Overlap of WT and R190H peaks. (C) Top 10 slimmed GO terms for targets of the A-, B- and C-sets from WT vs R189G. (D) Top 10 slimmed GO terms for targets of the A-, B- and C-sets from WT vs. R190H. (E) Overlap of genes lost (A-sets) by both backbone-contacting variants, from which a high-confidence STRING network was formed with 706 overlapping genes, showing highly significant network p-value, with cardiac network hubs highlighted in red. (F) Overlap of genes retained (B-sets) by both backbone-contacting variants, from which a high-confidence STRING network was formed with 349 overlapping genes, showing highly significant network p-value, with cardiac network hubs highlighted in red. (G) Overlap of genes gained (C-sets) by both YRD variants, from which a high-confidence STRING network was formed with 50 overlapping genes, showing no significant network enrichment or key hubs. (H) Significant Trawler de novo enriched motifs clustered to reduce redundancy for A-, B- and C-sets of R189G variant. (I) Significant Trawler de novo enriched motifs clustered to reduce redundancy for A-, B- and C-sets of R190H variant. (J and K) Counts of significantly bound 8-mers (E > 0.35) from PBM, for WT (dark blue) R189G (J) and R190H (K) (light blue). (L and M) Interaction patterns of R189G (L) and R190H (M) variants with cardiac TFs Nkx2-5, Gata4, Tbx5, Tbx20 and Hand1, using a Yeast-two-hybrid (Y2H) protein-protein interaction assay. Y2H fluorescence intensities were normalised to Nkx2-5 WT at 2 different time-points (T_1_ and T_2_) and represented as normal, perturbed or absent interaction plots.

We next focussed on CHD variants in the NKX2-5 HD affecting residues that normally contact the DNA backbone phosphates, namely R189G and R190H. As mentioned, the R190H variant is a true backbone-contacting variant, whereas R189G appears in the NKX2-5 crystal structural analysis as a “next to backbone-contacting” residue. However, their effects on NKX2-5 function appeared highly similar over multiple assays and it is possible that in 3D chromatin space, R189 is able to contact DNA-backbone phosphates; thus, for simplicity here we have designated both as *backbone-contacting*. Compared to YRD and other variants, these backbone-contacting variants showed greater severity of regulatory impact of any functional class. This was demonstrated by greater loss of NKX WT targets (increased A-sets; 82% and 63%, respectively), smaller B-sets (retained targets) and negligible C-sets (Figure 4A–B). As for YRD variants, GO term enrichment analysis using CompGO showed highly significant *muscle* and *actin cytoskeleton*-related signatures in A- and B-sets (Figure S8A–D). In addition, in the A-set, there was enrichment for terms related to more peripheral aspects of the NKX2-5 regulatory network governing *metabolic* and *catabolic* processes, *protein translation*, *dephosphorylation*, *apoptosis* and *cellular morphogenesis* (Figure 4C–D and S8C–D). Overlap of backbone-contacting variant A-sets revealed >85% intersection, again suggesting a shared mechanism for target loss within this functional class of variant (Figure 4E). Observationally, overlapping the A-set genes of backbone-contacting variants versus those of other functional classes showed a much lower concordance (between 35–55% overlap compared with 70–85%, within functional classes), highlighting that the differences in target loss between classes is network driven. PPI analyses using STRING highlighted loss of many fewer cardiac-related gene nodes in A-sets for backbone-contacting variants compared to YRD variants, and a shift towards loss of more peripheral NKX2-5 network genes involved in processes such as *RNA metabolism*, *insulin signalling* and *apoptosis* (Figure 4E). B-set target genes were predominantly linked to *muscle development* and *actin filament movement* in both GO term analyses (Figure 4C–D and S8C–D) and network analyses (Figure 4F). Thus, as for YRD variants, these backbone-contacting variants maintained an ability to bind muscle-related genes (i.e. elements of the core network), despite broad loss of targets. This was supported by pronounced over-representation of the NK2-class and NF-1 motifs in A- and B-set peaks (Figure 4H–I and S8E–H). Lastly, off-target C-set genes of these backbone-contacting variants, while overlapping, were highly distinct from one another by GO term analysis (Figure 4C–D and S8C–D) and displayed sparse connectivity (Figure 4G). This may be influenced by the small size of their C-set (Table S2). Corroborating previous findings using EMSA^117^, PBM demonstrated that the two backbone contacting variants showed diminished DNA-binding affinity (Figure 4J–K and S5C–D). This loss potentially stems from a compromised structural stability or altered conformation of the HD, and loss of non-specific interactions with DNA^62,118^. Y2H analysis revealed that R189G and R190H variants both showed disrupted interactions with NKX2-5 WT and all cardiac co-factors, differing only in their interactions with GATA4 (Figure 4L–M and S8I). Combined with analysis of YRD variants, these data suggest the profound involvement of both the HD and YRD in homodimerization with NKX2-5 and heterodimerization with all cardiac cofactors tested, with the exception of TBX5 which primarily interacts with the HD.

### Homeodomain DNA-base-contacting variants gain cardiac targets through enhanced cooperativity with cardiac TFs and altered DNA binding

**Figure 5.**
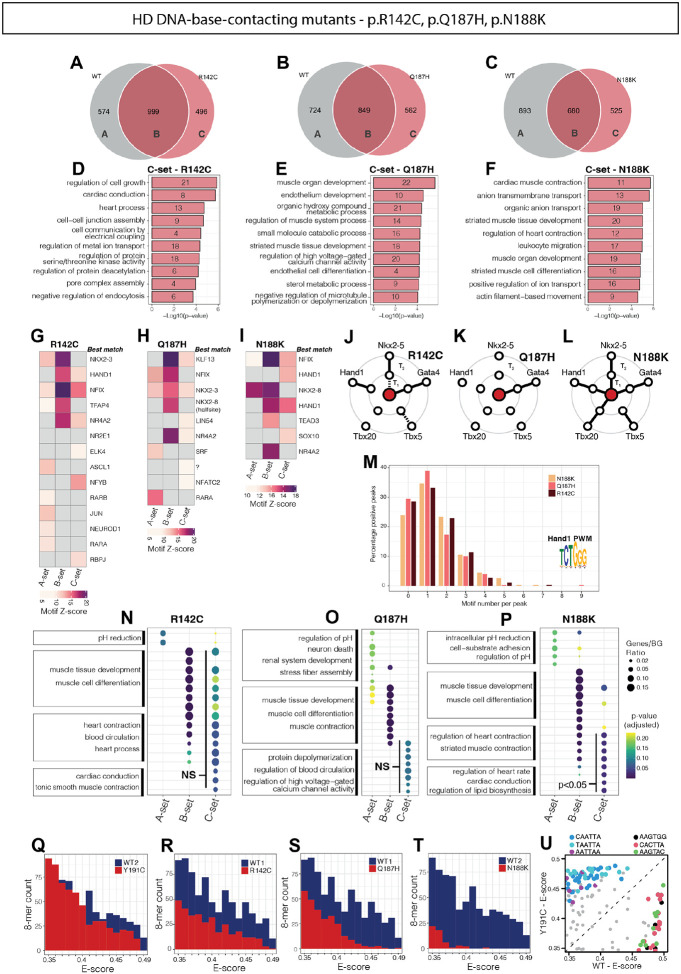
HD DNA-base-contacting variants are redistributed to ectopic cardiac targets via atypical interactions with cardiac TFs (A) Overlap of WT and R142C peaks. (B) Overlap of WT and Q187H peaks. (C) Overlap of WT and N188K peaks. (D) Top 10 slimmed GO terms for targets of the R142C C-set only. (E) Top 10 slimmed GO terms for targets of the Q187H C-set only. (F) Top 10 slimmed GO terms for targets of the N188K C-set only. (G) Significant Trawler de novo enriched motifs clustered to reduce redundancy for A-, B- and C-sets of R142C variant. (H) Significant Trawler de novo enriched motifs clustered to reduce redundancy for A-, B- and C-sets of Q187H variant. (I) Significant Trawler de novo enriched motifs clustered to reduce redundancy for A-, B- and C-sets of N188K variant. (J-L) Interaction patterns of R142C (J), Q187H (K) and N188K (L) variants with cardiac TFs Nkx2-5, Gata4, Tbx5, Tbx20 and Hand1, using a Yeast-two-hybrid (Y2H) protein-protein interaction assay. Y2H fluorescence intensities were normalised to Nkx2-5 WT at 2 different time-points (T_1_ and T_2_) and represented as normal, perturbed or absent interaction plots. (M) Count distribution of Hand1 motifs in C-set peaks of major base-contacting variants. (N-P) GO term analysis of genes associated with high Hand1 motif content peaks (3+ motifs per peak) in base-contacting variant A-, B- and C-sets of R142C (N), Q187H (O) and N188K (P), with C-set terms showing significance only for N188K. (Q-T) Counts of significantly bound 8-mers (E > 0.35) from PBM, for WT (dark blue) and base-contacting variants (red), Y191C (Q), R142C (R), Q187H (S) and N188K (T). (U) Sequence specificity of significantly bound 8-mers (E > 0.35) from PBM, for WT against Y191C, showing a change in specificity with Y191C.

The remaining functional class analysed were those NKX2-5 variants that changed DNA base-contacting residues. Among them, R142, N188, and Y191 are known to form hydrogen bonds with DNA bases, whereas Q187 makes weaker van der Waals contacts^89^. It was speculated that residue I184 weakly interacts with DNA bases; however, the evidence for this was not strong and it alternatively may function in between a HD-stabilising and DNA-base contacting residue, although this was not further interrogated^89^. Here, I184M was not analysed in depth. All base-contacting residues except R142 are situated within the third alpha helix of the NKX2-5 HD (Figure 1B). In base-contacting variants, there was a substantial loss of WT targets (A-sets), although less so compared to DNA backbone contacting variants, with N188K exhibiting the greatest loss (Figure 5A–C). However, the base contacting variant target genes overall maintained a relatively higher GO term similarity to WT as measured by CompGO (R=0.69–0.85; Figure S9A–C). Lost targets (A-sets) were associated with *actin filament organisation* terms but predominantly with more peripheral network processes such as *catabolism*, *apoptosis*, *regulation of kinase activity* and *dephosphorylation*, and *translation*, similar to backbone-contacting variant A-sets (Figure S9D–F). The relatively high number of retained (B-set) genes were consistently associated with *muscle* and *cardiac* GO terms, and these had much higher p-values than for A-set gene terms (Figure S9D–F), the same pattern as observed across other variant functional classes (Figure 3A–D and 5A–C).

What most distinguished base-contacting variants from other classes was their binding to a higher number of off-targets (C-sets) (Figure 5A–C). The C-sets for R142C, Q187H, and N188K represented 33%, 40% and 40% of total targets, respectively. Furthermore, unlike for other variant classes, genes associated with base-contacting C-set targets were uniquely enriched for a diverse array of distinct cardiomyocyte process, including *muscle development* and *differentiation*, *contraction* and *electrical coupling*, as well as others heart-related processes such as *endothelial development* and *metabolism* (Figure 5D–F). TF genes such as *Sox6*, *Nr2f2*, *Smad7*, *Hdac7*, *Hdac9*, *Fos*, *Mef2a*, *Etv1/2*, as well as various ion channel and intercellular junction genes contributed to the cardiac and endothelial C-set GO terms (Figure S10A–C). N188K was noteworthy in that it bound to targets associated with different membrane transporters, many of which are members of the solute carrier (*Slc*) gene family implicated in transport of organic ions and a variety of small molecules including amino acids, neurotransmitters and vitamins^119^ (Figure S10C). Similarly to both YRD and backbone-contacting variant classes, the A- and B-sets of base-contacting variants showed a high degree of overlap, highlighting again the shared mechanisms for target loss and retention in this class (Figure S9H–J). However, despite sharing muscle-related terms, base-contacting C-sets overlapped poorly (like other functional classes), suggesting that the mechanism by which this occurs is stochastic or highly dependent on the specific residue affected. These data together suggest a specific mechanistic rationale for the selection of unique off-targets by base-contacting variants.

We addressed whether these signatures involved changes in DNA-binding specificity, as shown for Y191C^33^. Analysis of *de novo* motifs using Trawler revealed that NKE and NF-1 motifs were highly over-represented in B-set peaks of all base-contacting variants (Figure 5G–I), signifying a conserved interaction with essential cardiac regulatory elements. Conversely, these motifs were less enriched in A- and C-sets. Enrichment analysis of *de novo* motifs with Homer (Figure S11A–C) and known motifs using Clover (Figure S11D–F) provided a similar picture, with the distinction that various NKE sites were enriched in almost all A-sets but absent in C-sets. Focusing on C-set off-target peaks, the most enriched *de novo* discovered motifs were for HAND, NF1, NFY, NR4, NKX2 and KLF TF families (Figure 5G–I). NFY family proteins are notable for their function as pioneer factors promoting chromatin accessibility for other TFs^120^. Known motif analysis revealed motifs for additional TFs in base-contacting C-sets, including GATA, SRF, MEF2, TAL1, p53, FOXO, MYB, STAT, AP1, EGR1 and MYOD families, some of which are known cofactors of NKX2-5^33–36,121,122^ (Figure S11A–D). Known motifs identified in base-contacting variant C-sets were also considerably more numerous than other variant functional classes, in-keeping with a unique type of gain-of-function.

Y2H analysis for cardiac co-factor interactions illustrated that each of the base-contacting variants presented a highly distinct pattern of interaction disruption when compared to WT (Figure 5J–L and S12). All variants maintained an interaction with GATA4, possibly clarifying the consistent presence of GATA motifs in both Clover analysis and Homer *de novo* enrichment, as well as the enrichment of cardiac targets in variant C-sets. Paradoxically, N188K, the base contacting variant most distinct from NKX2-5 WT (largest A-set and smallest B-set), maintained robust interactions with all tested co-factors (weakly for TBX5). Furthermore, this variant showed a particularly strong and persistent interaction with HAND1 at both days 1 and 2, which may account for the notable enrichment of HAND1 motifs within its C-set compared to A-set and B-set peaks. Because of this strong binding, we extended the Y2H assay out to day 5, observing a sustained interaction between N188K and HAND1, a pattern not observed for the interaction between HAND1 and NKX2-5 WT or other base-contacting variants, which ceased in our assay after day 2 (Figure S12). Thus, N188K can be characterised as a super-interactor with HAND1.

Investigating the functional outcomes of this HAND1 super-interaction with N188K *in vivo*, we found that total N188K DamID peaks displayed a slight preference for those with higher HAND1 motif content (4 or more HAND1 motifs per peak), compared with other base-contacting variants, however, this was not significant (Figure 5M). Trawler *de novo* motif enrichment and Clover known motif enrichment highlighted HAND1 motifs significantly enriched in N188K C-set peaks (Figure 5I and S10C,F). We filtered A-, B- and C-set peaks base-contacting variants that contained three or more HAND1 motifs, and conducted GO term analysis on their associated genes. For the purposes of this exercise, such targets are assumed to be either canonical HAND1 targets or co-bound NKX2-5-HAND1 sites. Analysis revealed that whereas filtered A-set genes show no significant association with cardiac GO terms, B-set genes of all base-contacting variants showed a strong association with cardiac GO terms, in-keeping with HAND1 being embedded in the NKX2-5 regulatory network at a high level^123^ (Figure 5N–P). Among filtered C-set genes, only those of the HAND super-interactor, N188K, showed significant enrichment of cardiac-related GO terms (adjusted p-value <0.05). Thus, the super-interaction between N188K and HAND1 provides a mechanistic rationale for an N188K gain-of-function, allowing binding to a unique set of peaks and genes associated with cardiac contraction and conduction, not bound by NKX2-5 WT or other base-contacting variants in HL1 cells.

Finally, PBM data revealed that base-contacting variants display varying degrees of loss of DNA-binding affinity from partial (R142C), to almost complete (N188K). Consistent with our previous DamID analysis of the HD specificity variant Y191C^33^, PBM analysis of this variant revealed a distinct shift in DNA binding specificity favouring a motif reminiscent of HOX proteins, however, without significant loss of affinity (Figure 5Q–U). A pattern of base-contacting variants progressively diverging from WT was seen here for 8-mer affinities (Figure 5R–T) and across all other analyses, highlighting the unique loss- and gain-of-target mechanisms at play within this functional class of mutation (Figure 1 and 2). These data together reinforce that C-set peaks can be selected via a specific regulatory logic involving atypical cofactor interactions, as previously demonstrated for an NKX2-5 mutation carrying a complete deletion of the HD, and/or by changes in DNA binding specificity^33^.

### Nuclear target search strategy is disrupted by HD DNA-contacting variants

**Figure 6.**
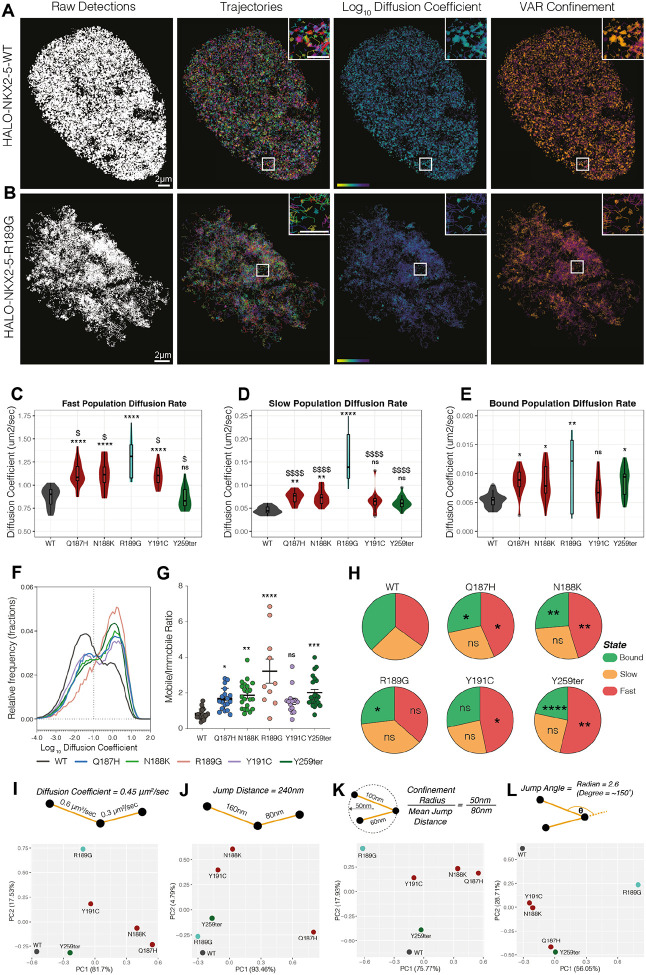
Single molecule tracking reveals divergent dynamics of DNA-base and DNA-backbone contacting NKX2-5 variants (A) Representative analysed super-resolution images of Halo-NKX2-5-WT and (B) Halo-NKX2-5-R189G mutant, showing tracked molecules from a single HeLa nucleus. Panels from left to right: 2D plots of all x,y,t single molecule detections (white), corresponding trajectories (arbitrarily coloured), trajectories colour-coded by log_10_ diffusion coefficient (yellow = lower values/mobilities; purple = higher values/mobilities), and trajectories colour-coded by confinement status as determined by vector autoregression (VAR) analysis (orange = confined; purple = unconfined). Panels in A and B were generated using a custom-made Python software (NASTIC). (C) Diffusion coefficient of fast-diffusing population of NKX2-5 WT or mutant molecules. (D) Diffusion coefficient of slow-diffusing population of NKX2-5 WT or mutant molecules. (E) Diffusion coefficient of stably bound population of NKX2-5 WT or mutant molecules. (F) Distribution of Log_10_ diffusion co-efficient for NKX2-5 WT and mutant molecules, split into mobile and immobile fractions, separated by dotted line. (G) Ratio of NKX2-5 WT or mutant molecules classified as mobile vs. immobile. (H) Percentages of trajectories categorised as either bound, slow- or fast-diffusing for NKX2-5 WT or mutant molecules. (I-L) Principal components analysis of molecular dynamics of NKX2-5 WT or mutant molecules analysed using TrackIt, showing diffusion coefficient per trajectory (I), mean jump distance per trajectory (J), confinement radius over mean jump distance per trajector (K) and jump angle per trajectory (L). Significance (C-E) was calculated with a Two-way ANOVA, * indicates difference from WT less than p<0.05, ** indicates difference from WT less than p<0.01 and **** indicates difference from WT less than p<0.0001. $ indicates difference from backbone-contacting mutants less than p<0.05 and. $$$$ indicates difference from backbonecontacting mutants less than p<0.0001.

Mammalian TFs spend >50% of their time searching chromatin for their functional DNA targets and their search efficiency is profoundly affected by chromatin structure and cofactor interactions^62^. To elucidate the chromatin binding and target search functions of NKX2-5 WT and its variants, we implemented a dual-modality single molecule tracking (SMT) imaging approach, similar to that of McCann and colleagues^79^. Briefly, this consisted of transient expression of NKX2-5 WT and variants N-terminally fused to the HaloTag self-labelling enzyme in Hela cells^124^. Following addition of the dye, JF549, which covalently binds to the HaloTag, we imaged single molecules using the super-resolution technique, highly inclined and laminated optical sheet super-resolution (HILO) microscopy^80,125^. The dual-modality imaging approach included capturing single molecules at two different acquisition rates: “fast” tracking at 20 msec intervals; and “slow” tracking at 500 msec intervals (Figure 6A–B and Figure S13). Each modality allows the determination of different molecular behaviours in real-time, with fast-tracking broadly capturing both mobile and immobile TF behaviours, and slow-tracking broadly capturing TF binding dynamics through the quantification of immobile molecule persistence (stable binding)^109^ (Figure S13 and S14). Fast-tracking data was analysed using a combination of tools (SLIMFast, SpotOn and TrackIt) to assess the presence of distinct molecular states, including spatiotemporal kinetics (Figure S13). Super-resolution images of tracked molecules in fast-tracking mode were also visualised using NASTIC^126^ (Figure 6A–B and S14). Slow-tracking data was analysed using SLIMFast^82^, which provided information on molecular binding dynamics and residence times (time spent in bound or immobile state) (Figure S13). We sought to assess the nuclear search dynamics of representative examples of the three key functional classes of NKX2-5 variants, namely, base-contacting, backbone-contacting and YRD variants. Previously characterised variant, Y191C, was included in this analysis as an example of a variant affecting DNA-binding specificity^33^.

Using SpotOn^84^ for fast tracking data, we segregated molecules into distinct populations or “states,” which included fast-moving, slow-moving, and bound, and calculated their average diffusion coefficients per cell (Figure 6C–E). Analysis revealed significant differences between WT and most variants for each state, with variant molecules generally exhibiting a higher diffusion coefficient than WT, with R189G the most severely affected. Y191C and Y259ter more closely resembled WT in molecular behaviours, their common feature being that they both have intact core HD folds. Further analysis using SLIMFast^82^ allowed us to plot total log diffusion coefficient curves, capturing the global behaviour of WT and its variants. Significant differences were evident between all variants and WT (Figure 6F–G). R189G, a severe-backbone contacting variant, displayed the most skewed log diffusion coefficient curve and, accordingly, mobile/immobile ratio (Figure 6A–G). This suggests that the R189G mutation severely disrupts the ability of NKX2-5 to efficiently search the nuclear space for its target sites. Log diffusion coefficient profiles for Y191C and Y259ter, with intact HD folds, were also highly skewed.

Differences in the proportion of molecules occupying each state were also determined (Figure 6H), with all variants spending more time in fast moving states and less time strongly bound. Intriguingly, R189G in this case was the least severely affected, whereas Y259ter exhibited the most substantial loss of strong binding state proportion, and the greatest increase in fast-moving state proportion, despite having an intact HD.

We next used the slow-tracking imaging modality (500 msec frame rate) to evaluate the residence times of NKX2-5 WT and variants in long- and short-lived dwell states, which represent binding to specific and nonspecific sites, respectively^85^. Dwell times are a function of the TF-DNA dissociation constant^62^. NKX2-5 WT showed average long- and short-lived dwell times of 5 sec and 0.9 sec, respectively (Figure S15A–B), comparable to other TFs involved in lineage specification^32^. Most variants exhibited a reduction in residence time by ~10–30%. Interestingly, the long-to-short lived ratio remained relatively constant, albeit that R189G, one of the most affected variants, showed a significant deviation (Figure S15C). This suggests that the residence times for short- and long-lived molecules are diminished roughly proportionally for most mutations. Cumulative decay function (CDF) analysis suggests that WT and variants have largely comparable turnover of bound molecules as a function of residence time, with only subtle differences (Figure S15D), except for R189G, which displayed a higher number of very long-lived molecules, an apparent gain-of-function (Figure S15D). This is surprising given the lower propensity of DNA backbone-contacting mutants to bind NKX2-5 WT targets, and suggests an alternate mechanism at play (Figure 4A–G).

Principal component analysis (PCA) of total trajectory length data derived from two consecutive windows of both fast- (20 msec) and slow-tracking (500 msec) modalities (i.e. how long a single trajectory lasted across the imaging window), indicated a very similar distribution of variants along the PC1 axis, which accounts for most variation (Figure S15E–F). Again, the backbone-contacting mutation, R189G, showed the greatest deviation from WT. Other base-contacting mutations showed similar, although less extreme, behaviours.

To deepen our understanding of the spatiotemporal dynamics exhibited by NKX2-5 WT and variants, we employed TrackIt^83^, a tool that generates detailed information on the 2D behaviour of single molecules. This analysis extended those using SpotOn and SLIMFast to include features of the spatial search space. It provided a comprehensive view, not only of how fast single NKX2-5 WT and variant molecules move, but how they interact spatially within the nuclear environment. PCA using TrackIt and diffusion coefficient probability plots confirmed that R189G exhibited the greatest deviation from WT, indicating enhanced nuclear mobility (Figure 6I and S16A), in line with findings above (Figure 6F–G). Conversely, Y191C and Y259ter, which have intact HDs, displayed the smallest deviation from WT. Other base-contacting mutants showed graded differences from NKX2-5 WT.

We next used TrackIt to analyse jump distances, a measure of how far a molecule travels between frames. Unlike the diffusion coefficient (Figure 6I), for jump distance PCA analysis showed that R189G clustered closely to WT (Figure 6J), however, it showed a markedly different jump distance distribution, strongly skewed towards larger distances (Figure S16B), likely because its diminished non-specific interactions with DNA provide less constraint^62,118^. All base-contacting variants and Y259ter (whose non-specific interactions with DNA are presumably intact) only mildly affected jump distance. We also analysed the molecule confinement radius, which reflects the geographic localisation of a molecule in a two-dimensional space. WT trajectories were highly confined, typically showing a confinement radius peaking at approximately 100 nm (Figure 6J), which is the approximate imaging pixel size (Figure 6A–B; Figure S16C), indicating that NKX2-5 WT has a highly restricted search space within the nucleus. In stark contrast, all variants showed profound effects on spatial confinement, as evidenced by PCA (Figure 6J), and for all mutants there was a clearly observable secondary peak of confinement radius peaking at around 400–500nm, not prominent for NKX2-5 WT (Figure S16C). R189G was most severely affected, with most ~100nm radius confinements lost. Thus, both non-specific (backbone-contacting) and specific (base-contacting residues) are important for achieving highly confined search volumes^32,62^.

Trajectory jump angles demonstrate the direction in which a molecule travels after each motion, in 360° search space. Jump angle analysis using PCA showed a distinct deviation from NKX2-5 WT for all variants (Figure 6L), with Y191C and N188K clustering tightly together, and Q187H and Y259ter also showing similar behaviour (Figure 6K). We analysed jump angle further, following a method similar to that of Hansen and colleagues^127^. We analysed the anisotropy of trajectories, focusing on connected trajectory segments with jump distances above 125nm, representing primarily freely-diffusing, non-bound tracks. This allowed us to determine whether there was a directional bias to nuclear movement for either WT or variant molecules. Indeed, we observed that NKX2-5 WT freely-diffusing molecules displayed significantly higher anisotropy compared to variants, peaking at 180° (Figure S17A–F). This indicates that WT molecules are substantially more likely to travel backwards than forwards after each movement, a directional propensity severely curtailed in variants. This distinctive behaviour suggests that NKX2-5 WT exhibits a specific nuclear search mechanism favouring anisotropic behaviour that is broadly lost in variants. The high anisotropy of jump distances >125nm was muted in trajectories <125nm, where all variants showed WT behaviour (Figure S17G–L).

Together, these analyses provide comprehensive insights into the altered chromatin search dynamics of NKX2-5 variants, underscoring significant changes in their movement, binding behaviour, and spatial dynamics within the nuclear environment. This offers, for the first time, insights into potentially diverse mechanisms of action for the effects of severe cardiac TF mutations on CHD.

## DISCUSSION

Since wide implementation of next generation sequencing and clinical testing, there has been an explosion of rare gene variants identified in children with CHD, many encoding TFs^3^. However, understanding CHD mechanisms and discovering targeted molecular therapies for survivors of CHD remains challenging, particularly as phenotypes arise from network perturbations at the earliest stages of heart development^37,39,57^.

We previously took an epigenomics approach to understanding CHD mechanism, profiling the genomic targets of NKX2-5 WT and a single natural disease-causing variant, Y191C^128^, as well as two synthetic mutations, one lacking the entire homeodomain (ΔHD)^33^. These mutations showed unique epigenomic signatures distinct from NKX2-5 WT that involved substantial loss and retention of WT targets, and gain of “off-targets”, via a regulatory logic related to altered DNA binding site specificity and/or changed protein-protein interactions (PPI)^33^. These findings suggest that a complex spectrum of loss, retention and unique gain-of-function effects underpin stably-expressed NKX2-5 disease-causing mutations.

Here, we undertook a broader functional-epigenomics approach to understanding these loss- and gain-of-functions in NKX2-5-related CHD. Our selection of variants allowed us to explore whether disruption of HD residues with known structural features or functions represent a convergent common class related to severe loss-of-function (haploinsufficiency^49^) or showed unique epigenomic impacts based on cognate functionalities. We analysed targets of twelve NKX2-5 variants affecting different types of residue within the HD, including DNA base-contacting, DNA backbone-contacting and HD stabilising residues, as well as other conserved PPI domains, in HL1 cardiomyocytes, as reported previously^33^. We combined this with deep analysis of DNA binding site specificity and affinity, co-factor PPI, and chromatin search and binding functions. Our results revealed a striking degree of diversity in NKX2-5 variant target signatures and cellular, biochemical and nuclear search behaviours, but also segregation into distinct functional classes, with class discrimination evident at single-residue resolution.

### General features:

All variants showed compelling lost (A-set), retained (B-set) and off-target (C-set) binding. The most severe variants (i.e. L171P, backbone-contacting variants [R189G and R190H] and YRD variants [Y25ter, Y>A]), showed greater overall loss of targets (increased A-sets combined with smaller B- and C-sets), compared to variants with milder impacts. A consistent feature of retained B-set target genes across all variants was the strong over-representation of cardiac muscle-related GO-terms and networks, indicating preservation of variant binding to core elements of the cardiac GRN, irrespective of severity. Significant shifts in the ratio of binding to NKX2-5 motifs of different affinities and specificity (viz. HOX-like motifs), as determined by PBM, appeared to be at least partially buffered *in vivo*, presumably though supportive co-factor and chromatin interactions. Lost targets (A-sets) showed muted association with cardiac muscle terms and were generally associated with more peripheral aspects of the cardiac GRN. Strikingly, A- and B-set genes individually showed high overlap when comparing variants linked by common structural and functional properties, i.e. backbone-contacting, base-contacting, and YRD variants, defining these as variant classes and proving a novel basis for consideration of CHD causation. C-sets showed lower degrees of overlap and higher p-values, suggesting higher stochasticity of off-target selection (Figure 5A–F).

### N-terminal variants:

N-terminal variants K15I and R25C lie respectively within, and adjacent to, the conserved N-terminal TN repressor domain (Figure 1A). Based on peak and GO term analyses, these variants appeared to be mildly affected (Figure 1C–D). K15I has been classified as *likely pathogenic* by ACMG, however, it shows weakly penetrant phenotypes and may act more as a genetic modifier^45,91^. Interestingly, K15I target genes showed a gain in the significance of muscle GO terms compared to WT (Figure 1C and E), consistent with loss of a repressive function.

### HD stabilising variants:

The two HD stabilising variants, T178M and L171P, showed strikingly different epigenomic signatures. T178M, despite being classified as *pathogenic* and associated with severe CHD in humans (Figure S1B), segmented closely with NKX2-5 WT in our analyses, as well as N-terminal, and other mild variants (Figure 1C–D). T178M also showed a mild reduction in DNA binding affinities to the NKE (Figure S5D), however, stronger deviation from WT was seen when considering the broader spectrum of related sequences (Figure 2E). Such changes can clearly be buffered *in vivo* andm in fact, for T178M are completely prevented, as PBM results showed profound effects on target identification, while DamID showed little *in vivo* binding differences (Figure 2E–G). In contrast, L171P, which introduces a kink into HD helix 2, breaking its continuity, leading to dominant-negative effects experimentally^100^, showed the greatest loss of targets and divergent GO term and motif signatures of all variants analysed. The epigenomic signatures of these two stabilising variants do not immediately suggest a mechanistic rationale for their divergent impacts (Figure S1B). Other features not studied here, such as cofactor interactions or search functions, may likely be involved and contribute to HD destabilising effects *in vivo*.

### HD DNA backbone-contacting variants:

Severe class-specific molecular phenotypes were seen for NKX2-5 DNA backbone-contacting variants R189G and R190H, likely because of their destabilising effects on HD structure and loss of non-specific binding to DNA^62,118^. Analysis revealed high overlap between R189G and R190H peaks overall, but poor overlap with NKX2-5 WT peaks, defining a mutation class (Figure 1C). The high overlap in individual A- and B-sets when comparing the two backbone-contacting variants strongly supports this notion. Molecular phenotypes for this class were among the most severe, however, in the context of only partial disruption of PPI with cofactors.

### DNA base-contacting variants:

Base-contacting variants, which contribute to DNA-binding specificity, showed graded degrees of relationship to NKX2-5 WT (Figure 1C; Figure S4A–B). A- and B-sets genes for different base-contacting variants showed a high degree of GO term concordance (Figure 5A–C; Figure S8H–J), thus defining an additional HD variant class. R142C is unusual for a base-contacting variant as it lies in the N-terminal tail of the HD, rather than the helices, interacting specifically with the DNA minor groove. Whereas it is predicted to be pathogenic, it segmented closely to NKX2-5 WT and other mildly affected variants and showed normal binding to the NKE 8-mer (Figure 1C–D; Figure S4A–B; Figure S5D). However, PPI with cardiac co-factor TBX20 was all but lost, and heavily disrupted for NKX2-5, HAND1 and TBX5; only a strong interaction to GATA4 was retained. R142C also showed reduced E-scores for binding across top 8-mers and a skewing of the proportion of binding to high versus low affinity sites, and HOX-related motifs, by PBM (Figure 2E and F; Figure 5R). As for other variants, R142C showed a gain in significance of cardiac muscle-related GO terms (Figure 1E), potentially indicating loss of repression. These collective features likely underlie its pathogenicity.

Most severe among base-contacting variants was N188K, almost as severe as backbone-contacting mutations (Figure 1C and D), however, displaying a distinct pathogenicity profile. N188K had among the most damaging impacts on binding affinity to the NKE and related lower affinity sites of all variants analysed (Figure S5D; Figure 2C–F; Figure 5T). Paradoxically, it retained PPI to NKX2-5 and cofactors with only a partial diminishment for TBX5 (which interacts with NKX2-5 via the HD^37^). Unique to variant classes analysed here, N188K C-set off-targets showed compelling over-representation of cardiac *muscle development*, *differentiation*, *contraction*, *actin filament* and *ion transport* terms and networks (Figure 5A–C; S9A–C). We discovered that N188K was in fact a super-interactor with the co-transcription factor HAND1, providing a likely mechanism for this effect. Accordingly, there was increased cardiac muscle GO-terms detected among all N188K gene targets and a highly significant over-representation of HAND motif clusters among C-set targets (Figure 1E and Figure 5I).

### YRD variants:

Variants within the conserved YRD represent yet another distinct variant class with severe molecular phenotypes. The YRD is a PPI domain which participates, along with the HD, in NKX2-5 homodimerization. It is also a heterodimerisation interface for co-factors ELK1 and ELK4, which are highly embedded within the cardiac network^33^. Our work here suggests that the YRD is also involved in PPI with GATA4, TBX20 and HAND1. We have previously shown that the YRD is essential for the function of NKX2-5 in early heart development^86^. Unique among variants described, YRD variants showed lost (A-set) target gene sets that were highly enriched for GO terms associated with *cardiac muscle development*, *differentiation* and *contraction* (a feature more typical of B-sets). Thus, YRD variants, despite having an intact HD and near-normal binding site affinity and specificity using PBM (Figure 3J–K and Figure S5D), failed to bind to large subsets of muscle-associated targets at the core of the cardiac GRN, as well as to more peripheral targets. The strong overlap between their A- and B-set target GO terms and gene networks (Figure 1C and Figure 3A–G), define YRD variants as a distinct class, separate to the HD variant classes described above. These data powerfully reinforce the importance of PPI in enhanceosome assembly and regulation of gene expression^33,37,57^, and the shared responsibility of the HD and the YRD for core NKX2-5 functions.

### Chromatin search and binding behaviours:

Mammalian TFs are believed to find their targets on chromatin through *facilitated diffusion* along DNA nano-fibres involving sliding, hopping and intersegmental transfer, nucleosome binding, condensate formation, and stepwise assembly of oligomeric complexes^62,118^. These TF search functions likely strike a balance between efficiently and comprehensively scanning DNA within diverse and changeable chromatin volumes, and exiting them to continue searching elsewhere. Over the last decade, single molecule tracking (SMT) has significantly advanced our understanding of these complex TF search functions, with different TFs displaying wide ranging chromatin search and binding behaviours. One implication is that TF amino acid sequences encode not only specificity and affinity parameters for functional DNA-binding and regulation of gene expression, but also chromatin search parameters, which may be profoundly affected by disease variants.

Here, we conducted a deep computational analysis of SMT data on NKX2-5 WT and select variants affecting base-contacting (Q187H, N188K), backbone-contacting (R189G), specificity (Y191C) and YRD residues, to explore how different functionalities impact chromatin search functions. All variants showed an increase in mean diffusion coefficients within their mobile, slow-moving and bound fractions, the latter two compartments thought to represent non-specific and specific DNA interactions with chromatin, respectively^62,118^ (Figure 6C–E and G–H). There was also a decrease in mean long-dwell and short-dwell residency times, reflecting an increase in decay rates and overall reduced DNA-binding time (Figure 6G and Figure S15A). Backbone-contacting variant R189G was the most severely affected, with base-contacting Q187H and N118K also showing strong deviation from WT. Interestingly, R189G uniquely showed a higher proportion of very long residency times (>20 sec), an apparent gain of function (Figure S15D). Overall, these findings concur with studies on a limited number of other TFs showing that non-specific DNA binding is potentially the more important feature of chromatin search functions and alone can lead to long-term residency events^32^. Non-specific interactions with chromatin are also a dominant feature of TF binding to mitotic chromosomes^129^. Non-specific chromatin interactions may involve direct binding to DNA and/or nucleosomes^32^. However, for some TFs, search functions have been found to be modulated by specificity residues or non-DNA-binding PPI determinants^32,85,130^. Our findings show that backbone-contacting, base-contacting, specificity and non-HD PPI residues profoundly participate in chromatin search functions by NKX2-5. These findings likely hold true for other HD subfamilies. The specific impacts of single variants (and variant classes) are rather similar compared to each other, and no individual variant showed catastrophic collapse. Based on theoretic grounds^118^, it seems probable that all variants studied here by SMT, even R198G, retain non-specific chromatin scanning capacity to some extent. Overall, our findings suggest that the scanning and specific target binding functions of NKX2-5 profoundly overlap and utilise the full extent of NKX2-5 functionalities, including the interdependent and separate roles of the HD and YRD. Indeed, TF binding sites detected using highly sensitive ChIP methods were found to include low affinity or degenerate binding sites^85^, which may be important for search functions.

PC analysis of search functions related to 3D confinement revealed highly divergent patterns for each HD variant (Figure 6I–L and Figure S15E–F), not unexpected given the divergent variant-specific signatures generated by DNA-binding, GO-term and PPI analyses. Deeper analysis confirmed R189G as the most severely affected (Figure S16A–C), and revealed that all HD and YRD variants frequently explored a large confinement radius (400–500nm) a behaviour not seen for NKX2-5 WT, likely reflecting altered search functions common to all classes.

### Limitations:

DamID data is collected from the mouse adult atrial HL1 cell line and will likely not capture NKX2-5 binding events relevant to all cardiac developmental and region-specific setting. Likewise, SMT data was collected in Hela cells in which cardiac-specific search or binding functions may vary. Given complex parameters influencing chromatin search functions and binding, and unique experimental settings, data may not be directly comparable between studies^62,118^. DamID and ChIP-related methods sample the genome in different ways and at different stoichiometries, and the outcomes are not directly comparable^33^. Differences likely relate in part to the different degrees to which each method samples non-specific and weakly-specific targets^85^. Bioinformatics processes are operator and software-dependent, and involved subjective decisions, as described previously^132^. The concepts and hypotheses underpinning this study, while based on human CHD-mutations and prior experimental data^33^, will require confirmation in more diverse settings, including in human tissues.

### Summary and perspective:

DamID has proven a highly discriminatory platform for NKX2-5 *in vivo* DNA binding signatures in cardiomyocytes. It has proven to be highly reproducible between NKX2-5 WT replicates performed years apart^34^ and, as seen here, between both mildly affected and WT variants, and class-specific severely-affected variants. It is important to note that “inappropriate” targets were also found for other CHD-causing TF variants using ChIP methods^37,57^, analogous to the off-target effects described here.

Our findings show highly individualised epigenomic and SMT signatures for each variant studied, which together impact a broad range of NKX2-5 functionalities. Data reveal that each variant has a unique combination of lost and retained targets, and unique off-targets. Our previous study revealed that when the NKX2-5 Y191C variant was expressed at near physiological levels in mouse ES cell-derived cardiomyocytes, the expression of many retained B-sets and off-targets were significantly altered, with most off-targets repressed^33^. Thus, altered expression of retained B-set target and off-targets have the potential to destabilise cardiac GRNs, a finding that can be generalised to other disease-causing TF mutations. C-set binding, as demonstrated here and previously^33^, can be underpinned by changes to DNA binding site specificity as well as the altered balanced of PPI, whereby mutant TFs can be hijacked to non-cardiac or additional cardiac sites. Retained B-set binding may result from the net effect of numerous cardiac-relevant cofactor interactions on variant binding; such targets may be part of super-enhancers that regulate core cardiac network genes^131^.

Despite the unique epigenomic signatures for each variant, we also detected class-specific behaviours related to DNA backbone-contacting, DNA base-contacting and YRD variants, which can be discriminated at single residue resolution. Variant classes relate to the distinct functionalities of participating residues. Class-specific epigenomic signatures may not translate cleanly into genotype-phenotype correlations in the clinic, as this may be masked by the overall impact of loss of function and increased network stochasticity common to severe variants. Yet overall, our study reveals the complex loss-of-function, retained function, and gain-of-function effects underpinning CHD. These findings can inform further work on understanding CHD mechanisms and future targeted molecular therapies in CHD patients.

## Supplementary Material

Supplement 1

## Data Availability

Data available upon request to corresponding authors
