# Supplementary material for: NKX2-5 congenital heart disease mutations show diverse loss and gain of epigenomic, biochemical and chromatin search functions underpinning pathogenicity
Source: bioRxiv. 2025 Jun 20:2025.06.20.659510. Preprint. [Version 1] doi: 10.1101/2025.06.20.659510 (PMC12191230; doi:10.1101/2025.06.20.659510)
Supplement: Supplement 1 [file media-1.pdf]

NKX2-5 congenital heart disease mutations show diverse loss and gain of epigenomic, biochemical and chromatin search functions underpinning pathogenicity

Alexander O. Ward[1,2,*], Nicole Schonrock[1,2], Alex J. McCann[3], Sabrina K. Phanor[4], Kian Hong Kock[4,5], Jesse V. Kurland[4], Fujian Wu[1,2], Nicholas J. Murray[1,2], James Walshe[6], Dimuthu Alankarage[1], Sally L Dunwoodie[1,2], Frederic A. Meunier[7], Mathias Francois[8], Martha L. Bulyk[4,5,9], Mirana Ramialison[10,11], Richard P. Harvey[1,2,12,*]

[1]Developmental and Stem Cell Biology Division, Victor Chang Cardiac Research Institute, Darlinghurst, Sydney, New South Wales, Australia

[2]School of Clinical Medicine, UNSW Sydney, Kensington, New South Wales, Australia

[3]Clem Jones Centre for Ageing Dementia Research, Queensland Brain Institute, The University of Queensland, St Lucia, Queensland, Australia

[4]Division of Genetics, Department of Medicine, Brigham and Women's Hospital and Harvard Medical School, Boston, Massachusetts, USA

[5]Program in Biological and Biomedical Sciences, Harvard University, Cambridge, Massachusetts, USA

[6]Department of Molecular Biology, Max Planck Institute, Gottingen, Germany

[7]School of Biomedical Science, The University of Queensland, St. Lucia, Queensland, Australia

[8]Centenary Institute, University of Sydney and Royal Prince Alfred Hospital, Sydney, New South Wales, Australia

[9]Department of Pathology, Brigham and Women's Hospital, and Harvard Medical School, Boston, Massachusetts, USA

[10]Australian Regenerative Medicine Institute, Monash University, Clayton, Victoria, Australia

[11]The Novo Nordisk Foundation Center for Stem Cell Medicine, reNEW Melbourne, Murdoch Children's Research Institute, Parkville, VIC, Australia; Department of Paediatrics, Faculty of Medicine, Dentistry and Health Sciences, University of Melbourne, Parkville, VIC, Australia

[12]School of Biotechnology and Biomolecular Science, UNSW Sydney, Kensington, New South Wales, Australia

***Corresponding authors.**

Alexander O. Ward (alex@tolemy.bio) and Richard P. Harvey (r.harvey@victorchang.edu.au)

SUPPLEMENTARY FIGURES

A

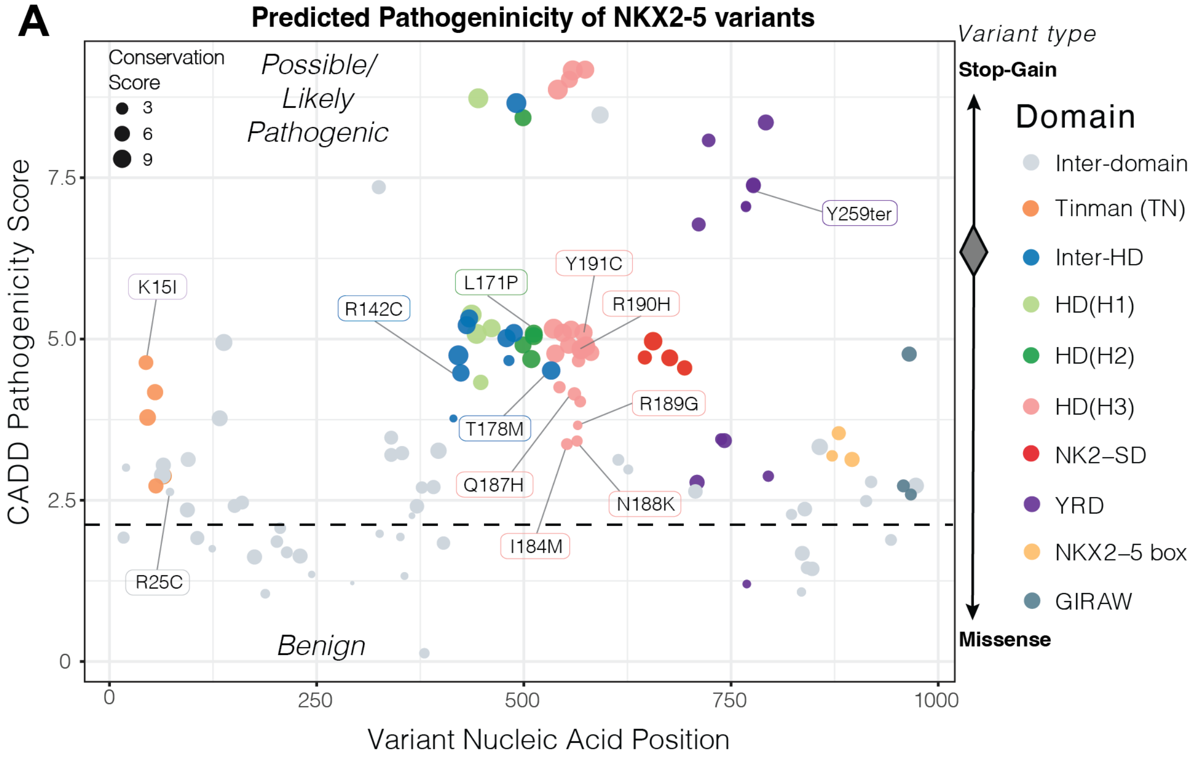

B

Mutation	Associated Phenotype	ACMG Classification	Domain +/- Residue Function
K15I	ASD	LP	***TN*** - Repressive*
R25C	ToF, HLHS, TD	VUS	***Downstream of TN*** - Repressive*
R142C	ASD, AVB, VSD, ToF, PDA, PS	P	***HD (N-terminal tail)*** - DNA-Base contacting
L171P	ASD, AVB, VSD, TVA	P	***HD (Helix II)*** - Stabilising/Structural
T178M	ASD, AVB, VSD, HLHS, SSS	P	***HD (Intra-Helix II-III)*** - Stabilising/Structural
I184M	ASD, TCA, CD, VNC, DCM, PFO	P	***HD (Helix III)*** - Stabilising/Structural
Q187H	ASD, AVB, AVR	P	***HD (Helix III)*** - DNA-Base-contacting
N188K	ASD, AVB, EA, TVA	P	***HD (Helix III)*** - DNA-Base-contacting
R189G	ASD, AVB, TVA, AF	P	***HD (Helix III)*** - Next to Backbone-contacting
R190H	ASD, AVB, VSD	P	***HD (Helix III)*** - DNA-Backbone-contacting
Y259ter	ASD, AVB, DORV, VSD	P	***YRD*** - Protein:Protein Interaction*
Y>A (x9)	Synthetic	N/A	***YRD*** - Protein:Protein Interaction*

C

Mutation	CADD	REVEL	BayesDel
**K15I**	4.63	0.79	0.4
**R25C**	2.63	0.352	-0.3
**R142C**	4.47	0.986	0.56
**L171P**	5.08	0.975	0.58
**T178M**	4.51	0.984	0.56
**I184M**	3.37	0.854	0.51
**Q187H**	4.15	0.958	0.54
**N188K**	3.44	0.922	0.26
**R189G**	3.16	0.938	0.53
**R190H**	4.85	0.985	0.57

Pathogenicity thresholds: CADD ≥ 2; Revel ≥ 0.6; BayesDel (maxAF) ≥ 0.1

Supplementary figure 1. Predicted pathogenicity of NKX2-5 variants is higher within known domains

(A) Predicted Pathogenicity scores (CADD) for all 141 *NKX2-5* variants, classified by domain. (B) Table showing variants assessed in this study, the associated phenotype, ACMG classification and the Domain and Residue affected by the variant. (C) Table showing pathogenicity scores as assessed by CADD, Revel and BayesDel.

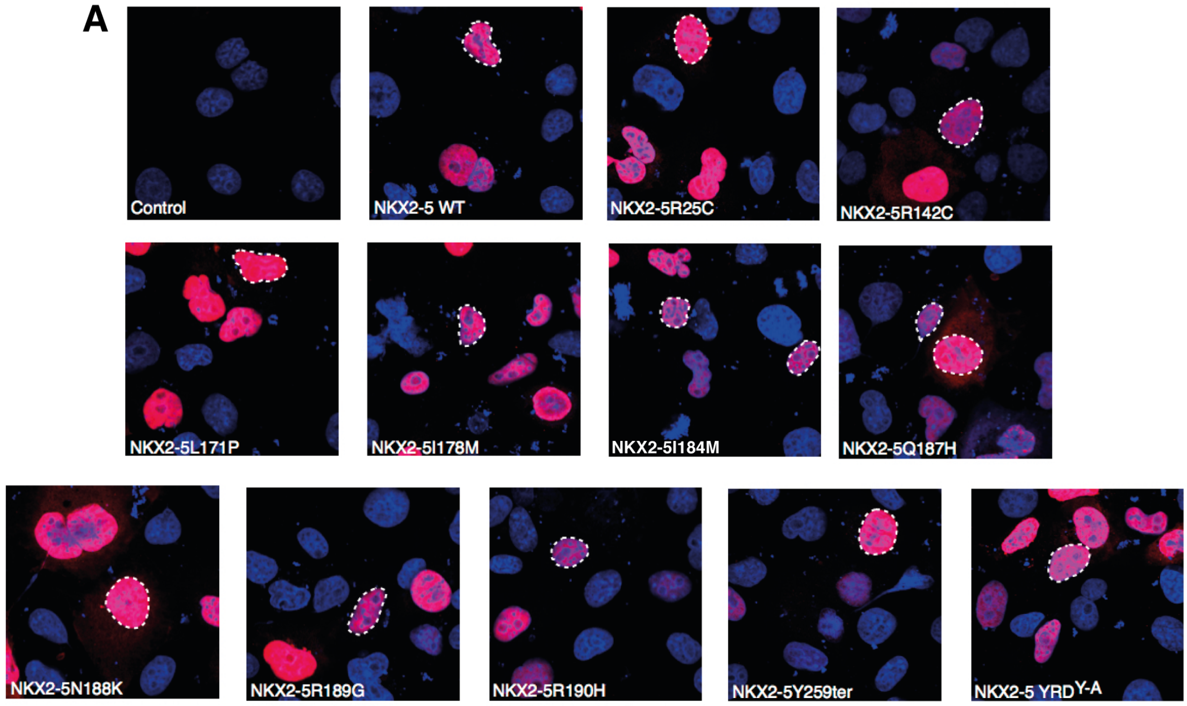

Supplementary figure 2. NKX2-5 WT and variants are all expressed in the nucleus

(A) Representative images of Cos-7 cells (lacking endogenous NKX2-5) transfected with WT and variant constructs, showing nuclear expression.

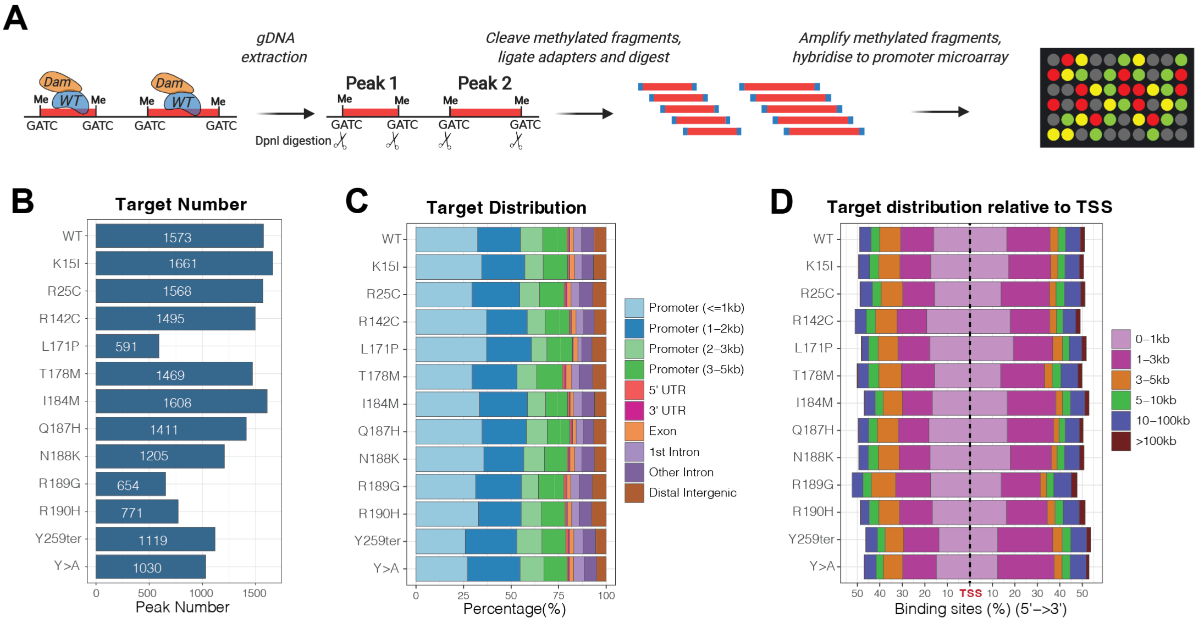

Supplementary figure 3. DamID-based approach to profile NKX2-5 WT and variant targets

(A) Schematic showing DamID approach and workflow used in this study. (B) Number of targets identified for WT and variants using DamID. (C) Genomic distribution of targets identified by DamID across known regions. (D) Distribution of targets identified by DamID relative to transcription start site.

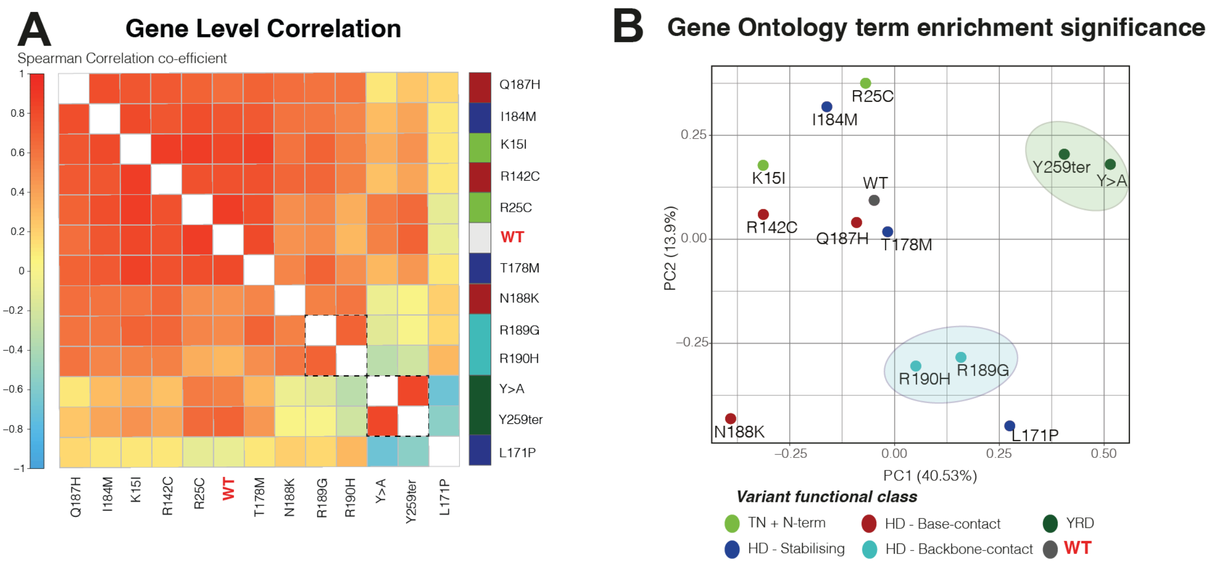

Supplementary figure 4. NKX2-5 variants further group by functional classes

(A) Spearman correlation between WT and variants at the level of target Genes identified.

(B) Gene Ontology terms clustered by enrichment significance levels in WT and variants.

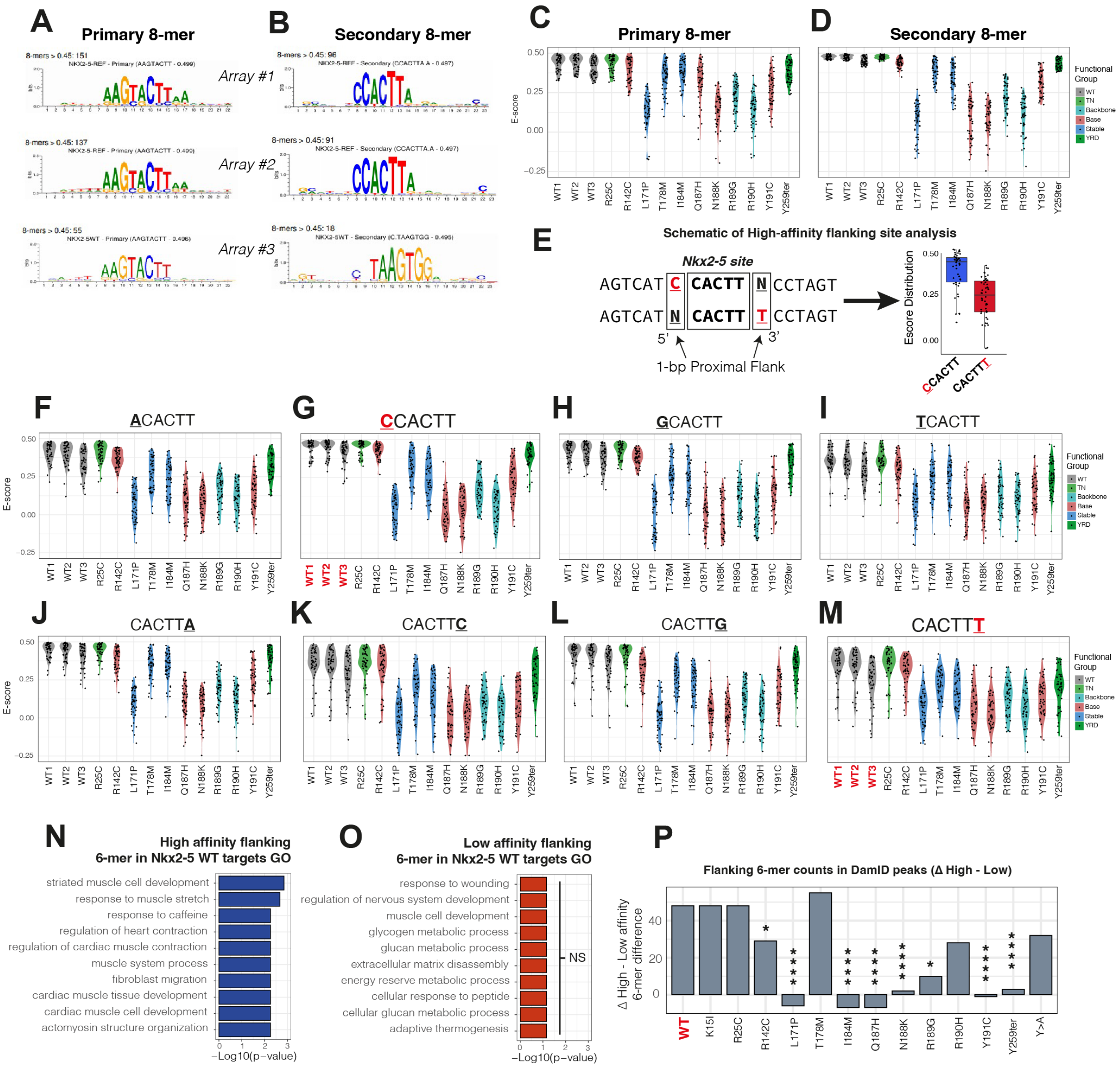

Supplementary figure 5. NKX2-5 variants show change cardiac regulatory logic

(A) Primary and (B) Secondary 8-mer identified by protein binding microarray (PBM), with the E-score distribut of top 50 (C) Primary and (D) Secondary 8-mers represented for three WT replicates and each variant. (E) Schematic of the custom flanking analysis performed. (F-M) E-score distribution of top 50 flanking 6-mers analysed with PBM, with high-affinity (CCACTT) and low-affinity (CACTTT) highlighted in red. (N) GO terms of WT DamID targets containing (N) high-affinity and (O) low-affinity 6-mers. (P) Delta in number of DamID peaks containing high- vs low-affinity peaks in WT and all variants.

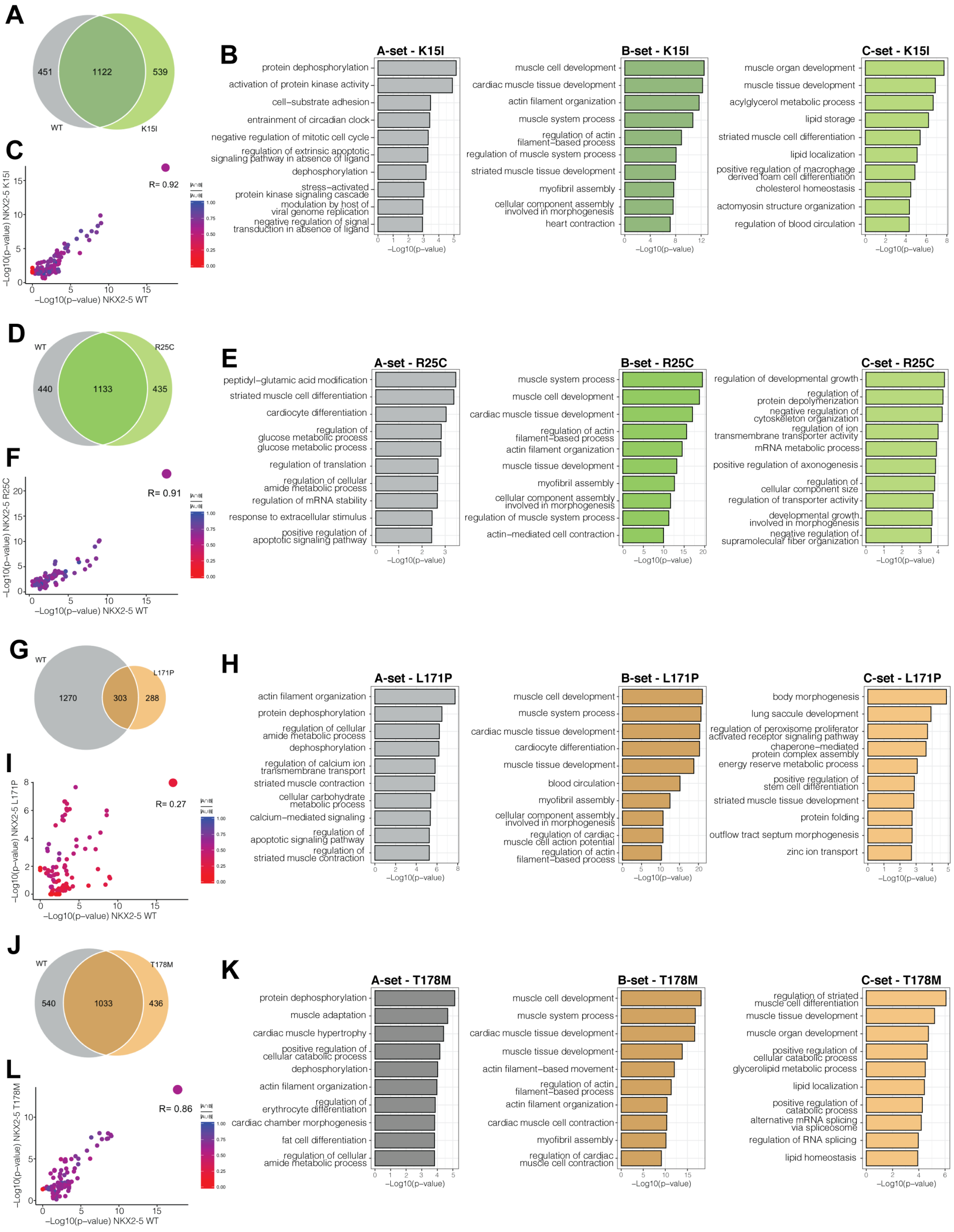
A
451
1122
539
WT
K15I
B
A-set - K15I
protein dephosphorylation
activation of protein kinase activity
cell−substrate adhesion
entrainment of circadian clock
negative regulation of mitotic cell cycle
regulation of extrinsic apoptotic signaling pathway in absence of ligand
dephosphorylation
stress−activated protein kinase signaling cascade
modulation by host of viral genome replication
negative regulation of signal transduction in absence of ligand
−Log10(p−value)
B-set - K15I
muscle cell development
cardiac muscle tissue development
actin filament organization
muscle system process
regulation of actin filament−based process
regulation of muscle system process
striated muscle tissue development
myofibril assembly
cellular component assembly involved in morphogenesis
heart contraction
−Log10(p−value)
C-set - K15I
muscle organ development
muscle tissue development
acylglycerol metabolic process
lipid storage
striated muscle cell differentiation
lipid localization
positive regulation of macrophage derived foam cell differentiation
cholesterol homeostasis
actomyosin structure organization
regulation of blood circulation
−Log10(p−value)
C
R= 0.92
−Log10(p−value) NKX2-5 K15I
−Log10(p−value) NKX2-5 WT
D
WT
R25C
440
1133
435
E
A-set - R25C
peptidyl−glutamic acid modification
striated muscle cell differentiation
cardiocyte differentiation
regulation of glucose metabolic process
glucose metabolic process
regulation of translation
regulation of cellular amide metabolic process
regulation of mRNA stability
response to extracellular stimulus
positive regulation of apoptotic signaling pathway
−Log10(p−value)
B-set - R25C
muscle system process
muscle cell development
cardiac muscle tissue development
regulation of actin filament−based process
actin filament organization
muscle tissue development
myofibril assembly
cellular component assembly involved in morphogenesis
regulation of muscle system process
actin−mediated cell contraction
−Log10(p−value)
C-set - R25C
regulation of developmental growth
regulation of protein depolymerization
negative regulation of cytoskeleton organization
regulation of ion transmembrane transporter activity
mRNA metabolic process
positive regulation of axonogenesis
regulation of cellular component size
regulation of transporter activity
developmental growth involved in morphogenesis
negative regulation of supramolecular fiber organization
−Log10(p−value)
F
R= 0.91
−Log10(p−value) NKX2-5 R25C
−Log10(p−value) NKX2-5 WT
G
WT
L171P
1270
303
288
H
A-set - L171P
actin filament organization
protein dephosphorylation
regulation of cellular amide metabolic process
dephosphorylation
regulation of calcium ion transmembrane transport
striated muscle contraction
cellular carbohydrate metabolic process
calcium−mediated signaling
regulation of apoptotic signaling pathway
regulation of striated muscle contraction
−Log10(p−value)
B-set - L171P
muscle cell development
muscle system process
cardiac muscle tissue development
cardiocyte differentiation
muscle tissue development
blood circulation
myofibril assembly
cellular component assembly involved in morphogenesis
regulation of cardiac muscle cell action potential
regulation of actin filament−based process
−Log10(p−value)
C-set - L171P
body morphogenesis
lung saccule development
regulation of peroxisome proliferator activated receptor signaling pathway
chaperone−mediated protein complex assembly
energy reserve metabolic process
positive regulation of stem cell differentiation
striated muscle tissue development
protein folding
outflow tract septum morphogenesis
zinc ion transport
−Log10(p−value)
I
R= 0.27
−Log10(p−value) NKX2-5 L171P
−Log10(p−value) NKX2-5 WT
J
WT
T178M
540
1033
436
K
A-set - T178M
protein dephosphorylation
muscle adaptation
cardiac muscle hypertrophy
positive regulation of cellular catabolic process
dephosphorylation
actin filament organization
regulation of erythrocyte differentiation
cardiac chamber morphogenesis
fat cell differentiation
regulation of cellular amide metabolic process
−Log10(p−value)
B-set - T178M
muscle cell development
muscle system process
cardiac muscle tissue development
muscle tissue development
actin filament−based movement
regulation of actin filament−based process
actin filament organization
cardiac muscle cell contraction
myofibril assembly
regulation of cardiac muscle cell contraction
−Log10(p−value)
C-set - T178M
regulation of striated muscle cell differentiation
muscle tissue development
muscle organ development
positive regulation of cellular catabolic process
glycerolipid metabolic process
lipid localization
positive regulation of catabolic process
alternative mRNA splicing via spliceosome
regulation of RNA splicing
lipid homeostasis
−Log10(p−value)
L
R= 0.86
−Log10(p−value) NKX2-5 T178M
−Log10(p−value) NKX2-5 WT

Supplementary figure 6. TN- and HD-Stabilising variants show highly divergent target binding

(A) Venn diagram with target overlap of WT and NKX2-5 K15I variant. (B) A-, B- and C-set gene ontology terms for NKX2-5 K15I variant. (C) Statistical GO-term analysis with CompGO comparing WT and NKX2-5 K15I variant. (D) Venn diagram with target overlap of WT and NKX2-5 R25C variant. (E) A-, B- and C-set gene ontology terms for NKX2-5 R25C variant. (F) Statistical GO-term analysis with CompGO comparing WT and NKX2-5 R25C variant. (G) Venn diagram with target overlap of WT and NKX2-5 L171P variant. (H) A-, B- and C-set gene ontology terms for NKX2-5 L171P variant. (I) Statistical GO-term analysis with CompGO comparing WT and NKX2-5 L171P variant. (J) Venn diagram with target overlap of WT and NKX2-5 T178M variant. (K) A-, B- and C-set gene ontology terms for NKX2-5 T178M variant. (L) Statistical GO-term analysis with CompGO comparing WT and NKX2-5 T178M variant.

Tyrosine Rich Domain (YRD) mutants - p.Y259ter & p.Y>A

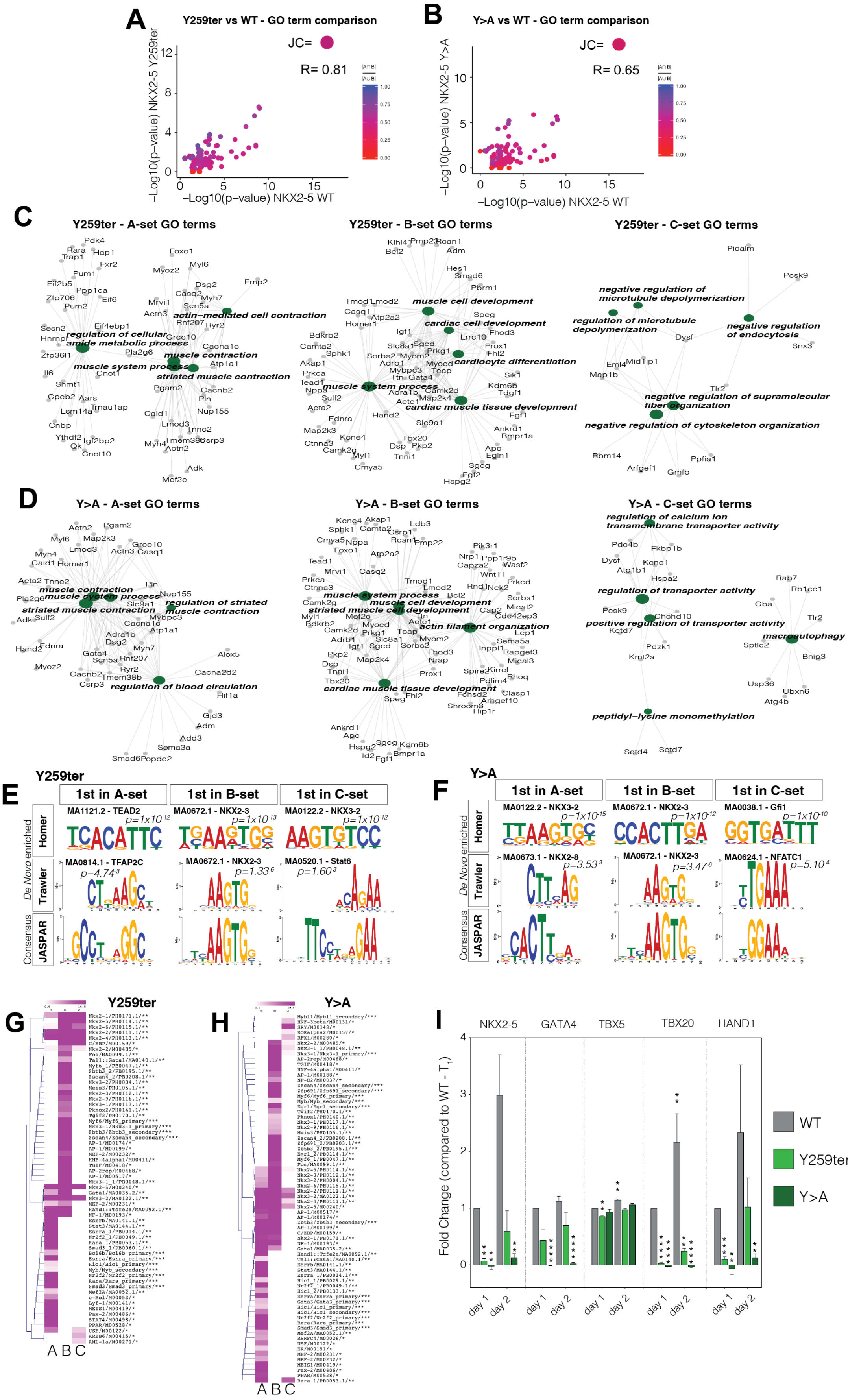

Supplementary figure 7. YRD variants have disrupted cardiac target binding

(A) Statistical GO-term analysis with CompGO comparing WT and NKX2-5 Y259ter variant. (B) Statistical GO-term analysis with CompGO comparing WT and NKX2-5 Y>A variant. GO-term Net Plot showing associated genes in the A, B and C-sets of (C) NKX2-5 Y259ter and (D) NKX2-5 Y>A. HOMER and Trawler motif analysis showing most enriched motif in the A, B and C-sets of (E) NKX2-5 Y259ter and (F) NKX2-5 Y>A. Clover known motif analysis showing most all identified motifs in the A, B and C-sets of (G) NKX2-5 Y259ter and (H) NKX2-5 Y>A. (I) Yeast-2-Hybrid analysis showing protein interaction with several cardiac TFs, shown as fold change from WT, for NKX2-5 Y259ter and NKX2-5 Y>A.

Homeodomain DNA-backbone-contacting variants - p.R189G & p.R190H

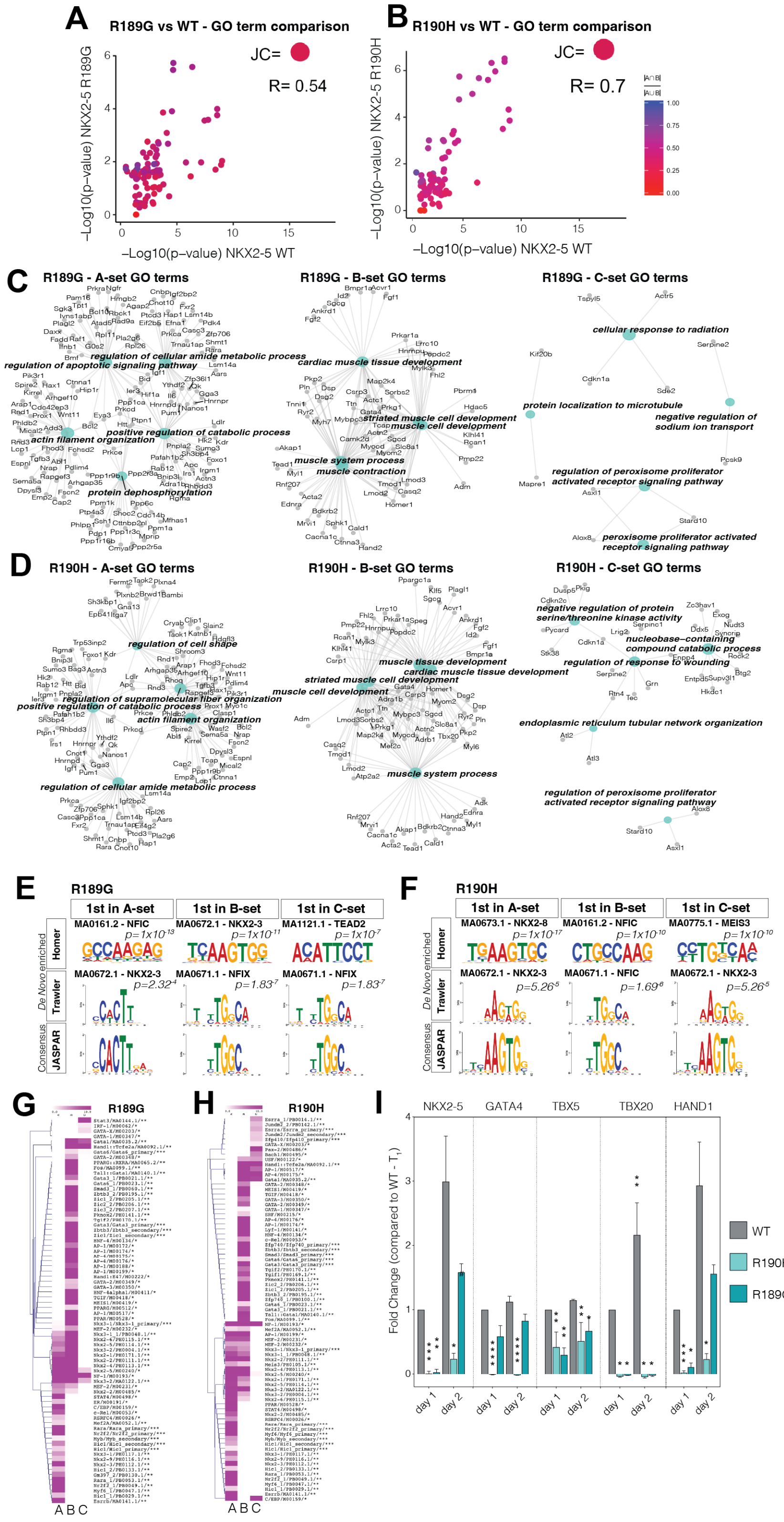

Supplementary figure 8. HD-DNA-backbone-contacting variants lose key cardiac targets

(A) Statistical GO-term analysis with CompGO comparing WT and NKX2-5 R189G variant. (B) Statistical GO-term analysis with CompGO comparing WT and NKX2-5 R190H variant. GO-term Net Plot showing associated genes in the A, B and C-sets of (C) NKX2-5 R189G and (D) NKX2-5 R190H. HOMER and Trawler motif analysis showing most enriched motif in the A, B and C-sets of (E) NKX2-5 R189G and (F) NKX2-5 R190H. Clover known motif analysis showing most all identified motifs in the A, B and C-sets of (G) NKX2-5 R189G and (H) NKX2-5 R189G. (I) Yeast-2-Hybrid analysis showing protein interaction with several cardiac TFs, shown as fold change from WT, for NKX2-5 R189G and NKX2-5 R190H.

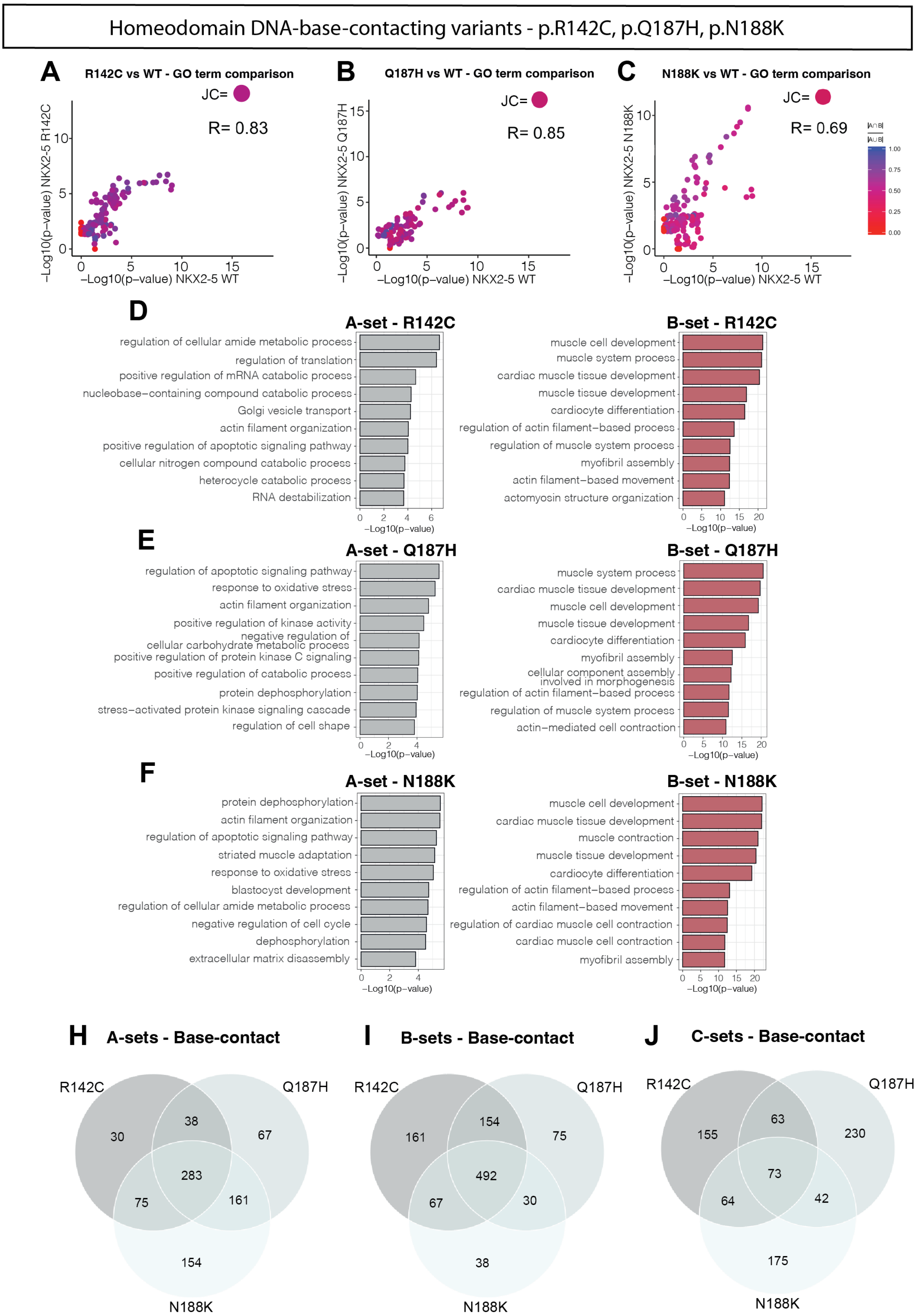

Homeodomain DNA-base-contacting variants - p.R142C, p.Q187H, p.N188K
A
R142C vs WT - GO term comparison
JC=
R= 0.83
−Log10(p−value) NKX2-5 R142C
−Log10(p−value) NKX2-5 WT
B
Q187H vs WT - GO term comparison
JC=
R= 0.85
−Log10(p−value) NKX2-5 Q187H
−Log10(p−value) NKX2-5 WT
C
N188K vs WT - GO term comparison
JC=
R= 0.69
−Log10(p−value) NKX2-5 N188K
−Log10(p−value) NKX2-5 WT
D
A-set - R142C
regulation of cellular amide metabolic process
regulation of translation
positive regulation of mRNA catabolic process
nucleobase−containing compound catabolic process
Golgi vesicle transport
actin filament organization
positive regulation of apoptotic signaling pathway
cellular nitrogen compound catabolic process
heterocycle catabolic process
RNA destabilization
−Log10(p−value)
B-set - R142C
muscle cell development
muscle system process
cardiac muscle tissue development
muscle tissue development
cardiocyte differentiation
regulation of actin filament−based process
regulation of muscle system process
myofibril assembly
actin filament−based movement
actomyosin structure organization
−Log10(p−value)
E
A-set - Q187H
regulation of apoptotic signaling pathway
response to oxidative stress
actin filament organization
positive regulation of kinase activity
negative regulation of cellular carbohydrate metabolic process
positive regulation of protein kinase C signaling
positive regulation of catabolic process
protein dephosphorylation
stress−activated protein kinase signaling cascade
regulation of cell shape
−Log10(p−value)
B-set - Q187H
muscle system process
cardiac muscle tissue development
muscle cell development
muscle tissue development
cardiocyte differentiation
myofibril assembly
cellular component assembly involved in morphogenesis
regulation of actin filament−based process
regulation of muscle system process
actin−mediated cell contraction
−Log10(p−value)
F
A-set - N188K
protein dephosphorylation
actin filament organization
regulation of apoptotic signaling pathway
striated muscle adaptation
response to oxidative stress
blastocyst development
regulation of cellular amide metabolic process
negative regulation of cell cycle
dephosphorylation
extracellular matrix disassembly
−Log10(p−value)
B-set - N188K
muscle cell development
cardiac muscle tissue development
muscle contraction
muscle tissue development
cardiocyte differentiation
regulation of actin filament−based process
actin filament−based movement
regulation of cardiac muscle cell contraction
cardiac muscle cell contraction
myofibril assembly
−Log10(p−value)
H
A-sets - Base-contact
R142C
Q187H
N188K
30
38
67
283
75
161
154
I
B-sets - Base-contact
R142C
Q187H
N188K
161
154
75
492
67
30
38
J
C-sets - Base-contact
R142C
Q187H
N188K
155
63
230
73
64
42
175

Supplementary figure 9. HD-DNA-base-contacting variants retain key cardiac targets

(A) Statistical GO-term analysis with CompGO comparing WT and NKX2-5 R142C variant. (B) Statistical GO-term analysis with CompGO comparing WT and NKX2-5 Q187H variant. (C) Statistical GO-term analysis with CompGO comparing WT and NKX2-5 N188K variant. Top 10 slimmed GO terms for A- and B-sets of (D) R142C, (E) Q187H and (F) N188K. Gene-level overlaps between all base-contacting variants, comparing (H) A-sets, (I) B-sets and (J) C-sets.

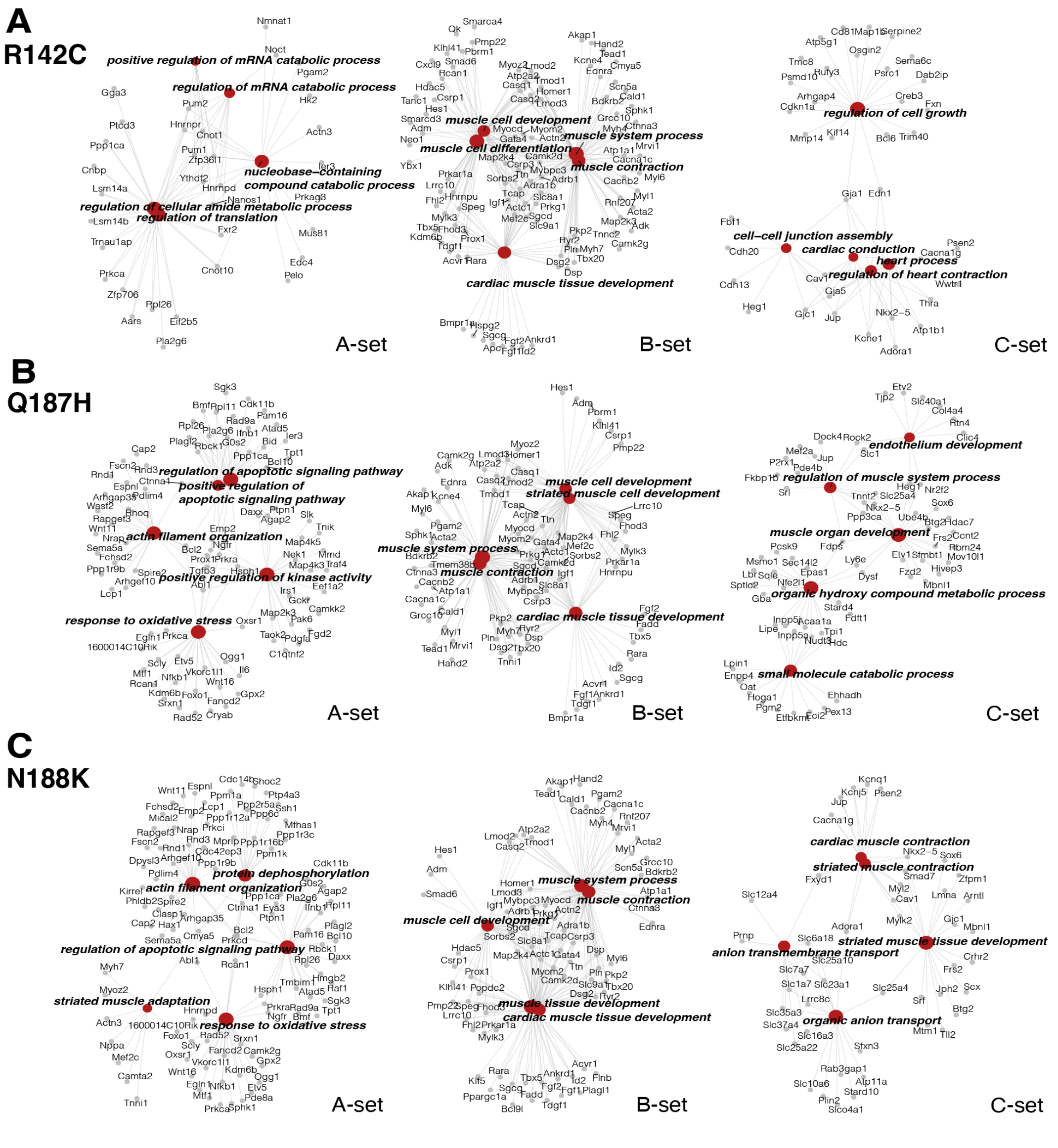

Supplementary figure 10. HD-DNA-base-contacting variants retain key cardiac targets

GO-term Net Plot showing associated genes in the A, B and C-sets of (A) NKX2-5 R142C, (B) Q187H and (C) NKX2-5 N188K.

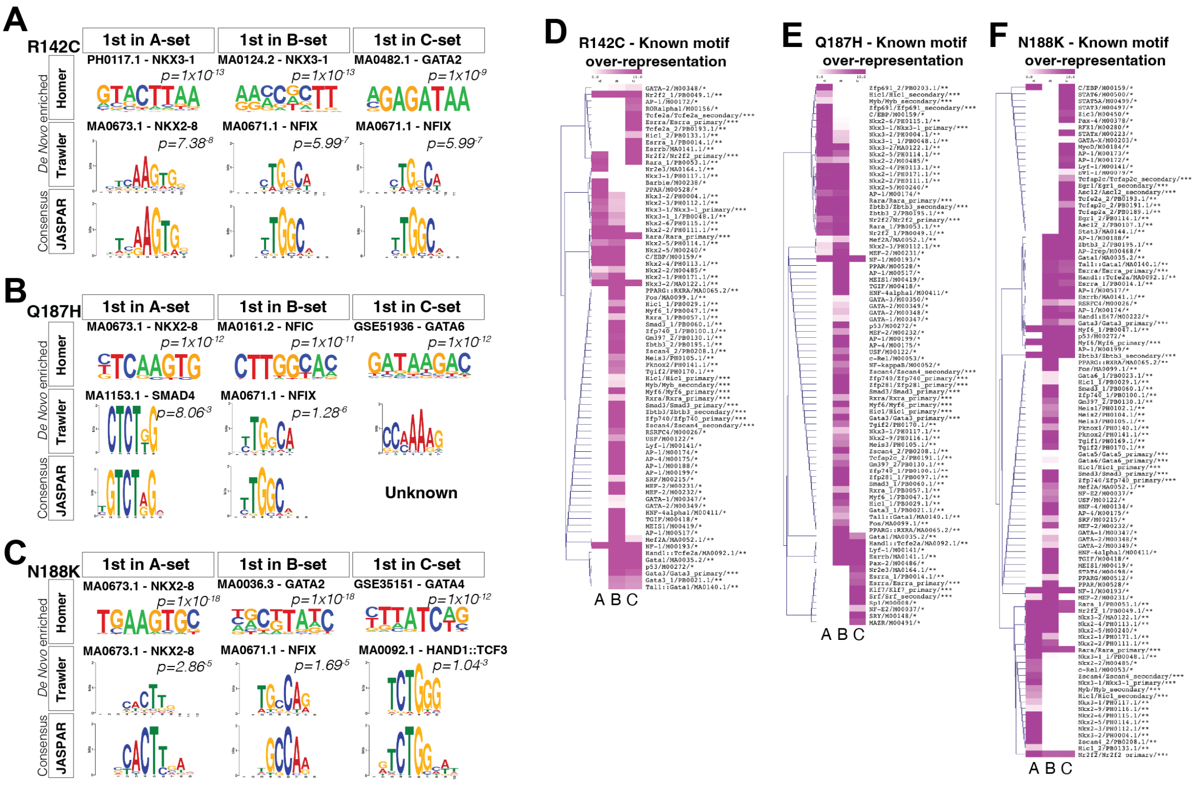

Supplementary figure 11. HD-DNA-base-contacting variants retain key cardiac targets

HOMER and Trawler motif analysis showing most enriched motif in the A, B and C-sets of (A) NKX2-5 R142C, (B) NKX2-5 Q187H and (C) NKX2-5 N188K. Clover known motif analysis showing most all identified motifs in the A, B and C-sets of (D) NKX2-5 R142C, (E) NKX2-5 Q187H and (F) NKX2-5 N188K.

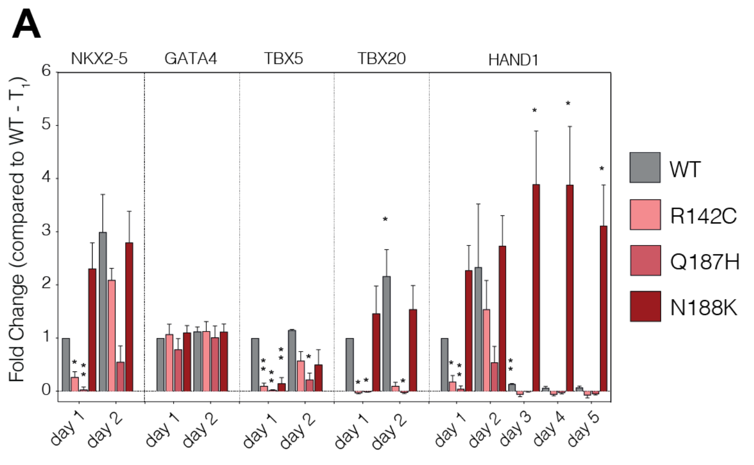

Supplementary figure 12. HD-DNA-base-contacting variants lose interaction with some cardiac TFs

(A) Yeast-2-Hybrid analysis showing protein interaction with several cardiac TFs, shown as fold change from WT, for NKX2-5 R142C, NKX2-5 Q187H and NKX2-5 N188K.

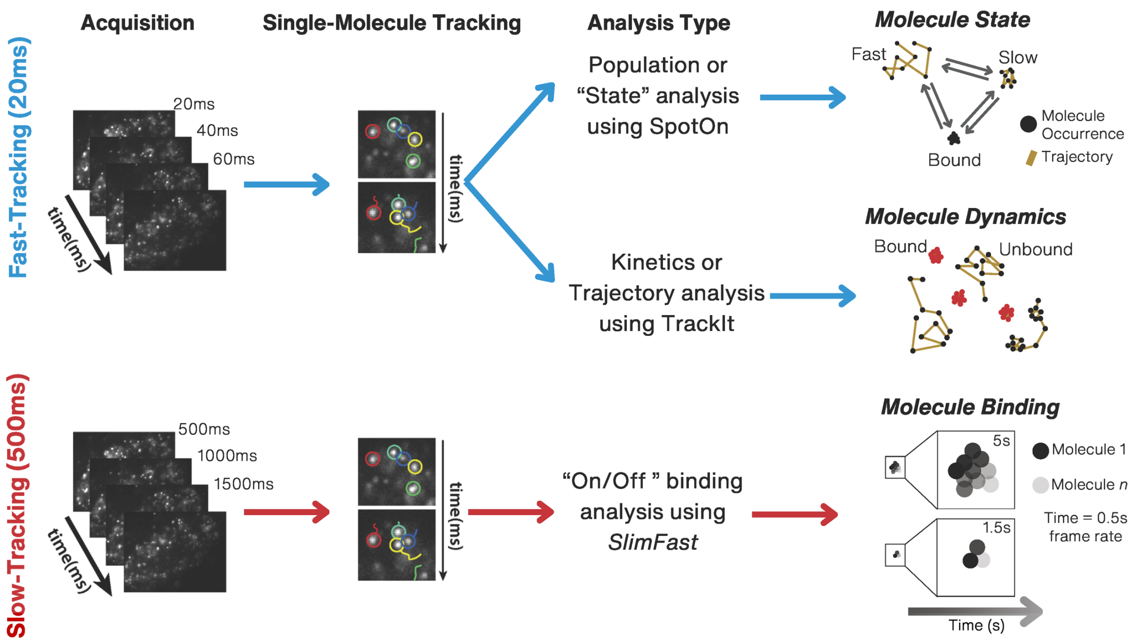

Supplementary figure 13. Schematic showing acquisition and analysis strategy for single molecule tracking

Full stepwise schematic showing microscopy and analysis approach for single molecule fast- and slow-tracking super-resolution microscopy.

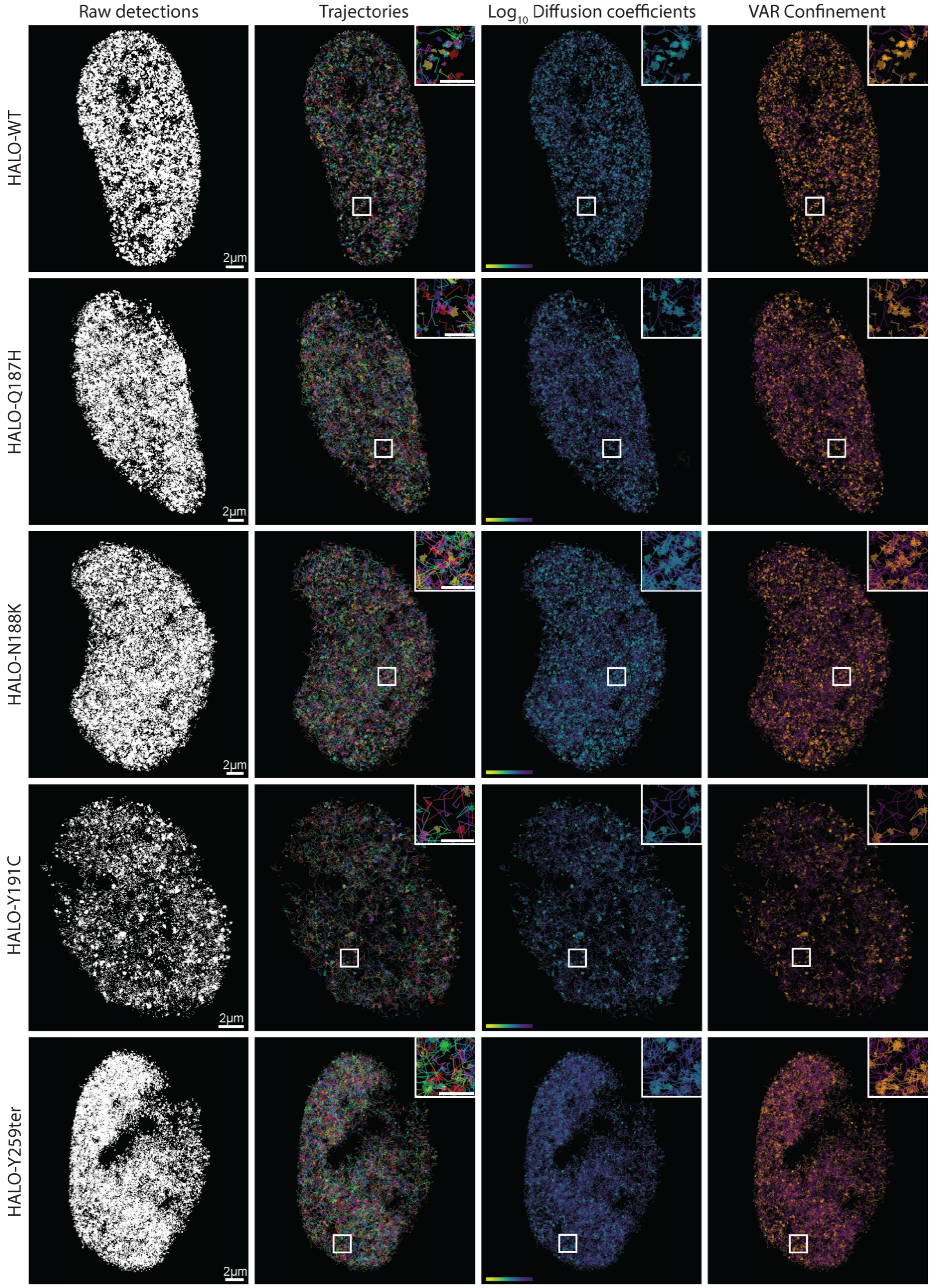
Raw detections
Trajectories
Log10 Diffusion coefficients
VAR Confinement
HALO-WT
HALO-Q187H
HALO-N188K
HALO-Y191C
HALO-Y259ter
2µm
2µm
2µm
2µm
2µm

Supplementary figure 14. Representative images from remaining single molecule tracking

Representative analysed super-resolution images of Halo-NKX2-5-WT and all remaining Halo-NKX2-5 variants showing tracked molecules from a single HeLa nucleus. Panels from left to right: 2D plots of all x,y,t single molecule detections (white), corresponding trajectories (arbitrarily coloured), trajectories colour-coded by $\log_{10}$ diffusion coefficient (yellow = lower values/mobilities; purple = higher values/mobilities), and trajectories colour-coded by confinement status as determined by vector autoregression (VAR) analysis (orange = confined; purple = unconfined). Panels in were generated using a custom-made Python software (NASTIC).

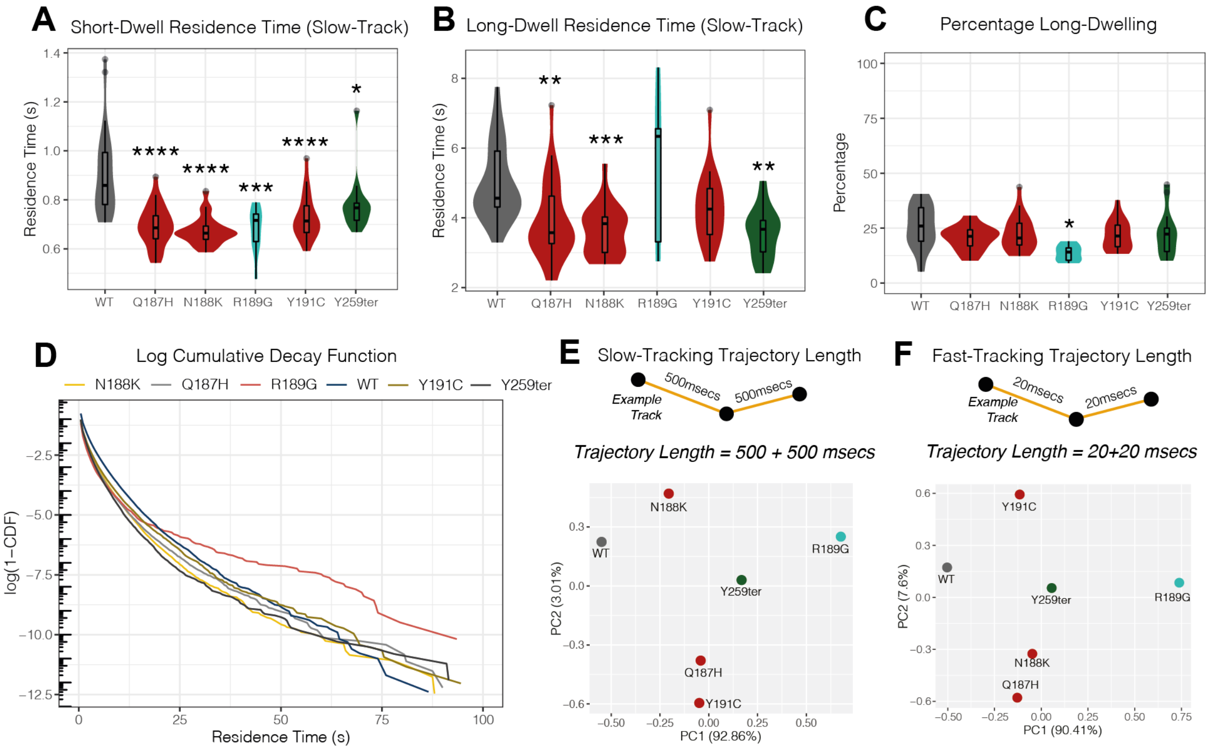

Supplementary Figure 15. NKX2-5 variants show reduction in both short-lived dwell and long-lived stable binding residence times

(A) Distribution of residence times for short-lived dwelling molecules, captured with slow-tracking approach. (B) Distribution of residence times for long-lived dwelling molecules, captured with slow-tracking approach. (C) Percentage of molecules classified as long-lived dwelling across NKX2-5 WT and variants. (D) Decay curves showing residence times of WT and variant molecules captured with slow-tracking. Principal Components Analysis of total trajectory lengths calculated using slow-tracking (E) and fast-tracking (F).

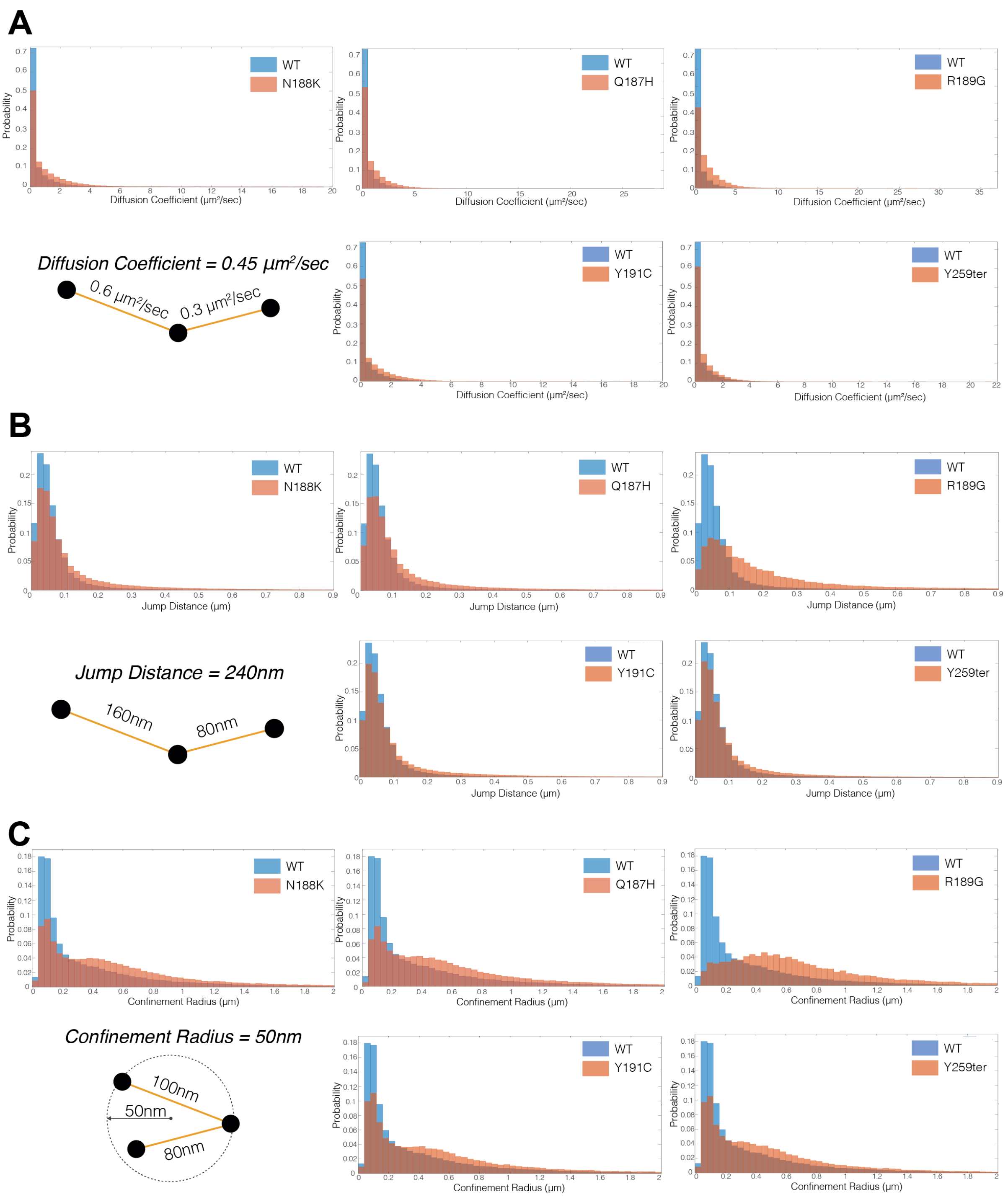

Supplementary Figure 16. NKX2-5 variants show broadly altered spatiotemporal dynamics

(A) Diffusion coefficient frequency (um2/sec) of all tracks from WT and variants, calculated using TrackIt. (B) Jump distance (um) frequency of all tracks from WT and variants, calculated using TrackIt. (C) Confinement radius (um) frequency of all tracks from WT and variants, calculated using TrackIt.

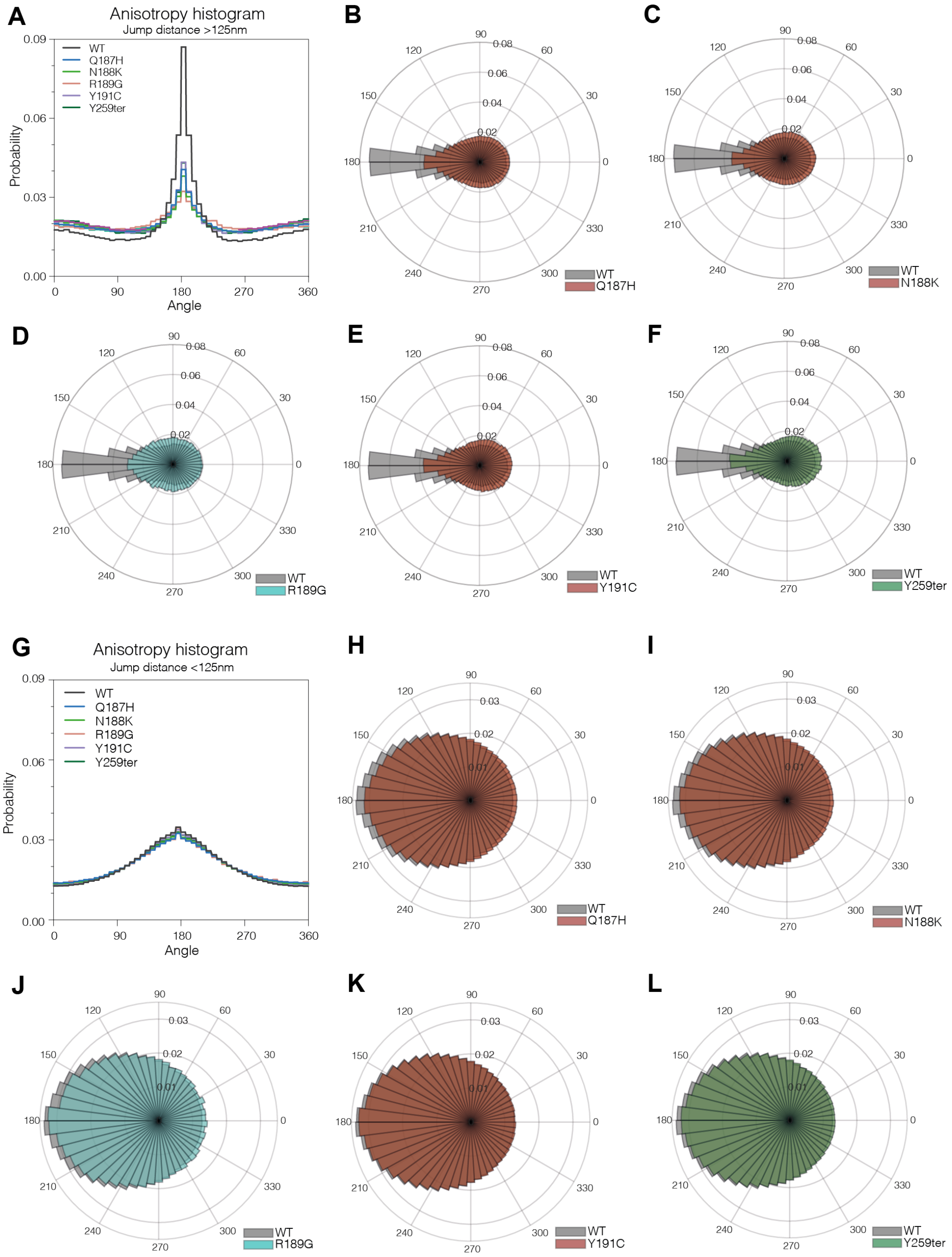

Supplementary Figure 17. Molecular trajectories of NKX2-5 WT are more anisotropic than variants

(A) Anisotropy Histogram of all trajectories that "jump" or move over 125nm between frames, for WT and all variants. (B-F) 360º Trajectory angles of all variants compared to NKX2-5 WT of all trajectories that "jump" or move over 125nm between frames. (G) Anisotropy Histogram of all trajectories that "jump" or less than 125nm between frames, for WT and all variants. (H-L) 360º Trajectory angles of all variants compared to NKX2-5 WT of all trajectories that "jump" or move less than 125nm between frames.

SUPPLEMENTARY TABLES

Mutation	Associated Phenotype	ACMG Classification	Domain +/- Residue Function
**K15I**	ASD	LP	***TN*** - Repressive*
**R25C**	ToF, HLHS, TD	VUS	***Downstream of TN*** - Repressive*
**R142C**	ASD, AVB, VSD, ToF, PDA, PS	P	***HD (N-terminal tail)*** - DNA-Base contacting
**L171P**	ASD, AVB, VSD, TVA	P	***HD (Helix II)*** - Stabilising/Structural
**T178M**	ASD, AVB, VSD, HLHS, SSS	P	***HD (Intra-Helix II-III)*** - Stabilising/Structural
**I184M**	ASD, TCA, CD, VNC, DCM, PFO	P	***HD (Helix III)*** - Stabilising/Structural
**Q187H**	ASD, AVB, AVR	P	***HD (Helix III)*** - DNA-Base-contacting
**N188K**	ASD, AVB, EA, TVA	P	***HD (Helix III)*** - DNA-Base-contacting
**R189G**	ASD, AVB, TVA, AF	P	***HD (Helix III)*** - Next to Backbone-contacting
**R190H**	ASD, AVB, VSD	P	***HD (Helix III)*** - DNA-Backbone-contacting
**Y259ter**	ASD, AVB, DORV, VSD	P	***YRD*** - Protein:Protein Interaction*
**Y>A (x9)**	Synthetic	N/A	***YRD*** - Protein:Protein Interaction*

Supplementary Table 1. **NKX2-5 mutations assayed by DamID, with associated phenotype and the affected residue function**

Mutation	A-set - Interaction Enrichment (# Nodes)	B-set - Interaction Enrichment (# Nodes)	C-set - Interaction Enrichment (# Nodes)
**K15I**	7.81e-5 (342)	<1e-16 (958)	*0.0668 (367)**
**R25C**	6.93e-5 (320)	<1e-16 (970)	0.00462 (239)
**R142C**	3.11e-7 (425)	<1e-16 (874)	*0.0583 (354)**
**L171P**	1.62e-11 (987)	<1e-16 (311)	*0.679 (202)**
**T178M**	0.000167 (394)	<1e-16 (906)	0.0216 (331)
**I184M**	9.14e-5 (428)	<1e-16 (872)	3.04e-6 (417)
**Q187H**	3.51e-7 (548)	<1e-16 (751)	0.0471 (407)
**N188K**	6.77e-5 (671)	<1e-16 (627)	3.53e-5 (354)
**R189G**	6.68e-11 (814)	<1e-16 (484)	*0.947 (86)**
**R190H**	2.88e-10 (839)	<1e-16 (459)	*0.0935 (174)**
**Y259ter**	<1e-16 (571)	1.88e-12 (729)	*0.233 (222)**
**Y>A (x9)**	<1e-16 (692)	7.22e-10 (607)	*0.205 (289)**

Supplementary Table 2. **STRING interaction enrichment p-values and gene-set sizes (Node) for A-, B- and C-sets for all NKX2-5 mutants**

Mutation	CADD	REVEL	BayesDel
**K15I**	4.63	0.79	0.4
**R25C**	2.63	0.352	-0.3
**R142C**	4.47	0.986	0.56
**L171P**	5.08	0.975	0.58
**T178M**	4.51	0.984	0.56
**I184M**	3.37	0.854	0.51
**Q187H**	4.15	0.958	0.54
**N188K**	3.44	0.922	0.26
**R189G**	3.16	0.938	0.53
**R190H**	4.85	0.985	0.57

Pathogenicity thresholds: CADD ≥ 2; Revel ≥ 0.6; BayesDel (maxAF) ≥ 0.1

Supplementary Table 3. **Pathogenicity scores for all NKX2-5 variants**
